# HSD17B7 Counters Bone Loss in Estrogen Deficiency via Estrogen Receptor Stabilization and Mediates the Effect of Raloxifene

**DOI:** 10.1002/mco2.70623

**Published:** 2026-01-31

**Authors:** Junyue Zhang, Yiping Song, Jeong‐Hyun Koo, Si Chen, Kyu Yun Jang, Sun‐Jung Yoon, Jung Ryul Kim, Young Jae Moon

**Affiliations:** ^1^ Department of Biochemistry and Molecular Biology Jeonbuk National University Medical School Jeonju Republic of Korea; ^2^ Department of Pathology Jeonbuk National University Medical School Jeonju Republic of Korea; ^3^ Department of Orthopaedic Surgery Jeonbuk National University Medical School Jeonju Republic of Korea; ^4^ Department of Orthopaedic Surgery Jeonbuk National University Hospital Jeonju Republic of Korea; ^5^ Research Institute of Clinical Medicine, Medical Sciences, and Biomedical Research Institute Jeonbuk National University and Hospital Jeonju Republic of Korea

**Keywords:** estrogen receptor alpha, HSD17B7, mitochondria, osteoporosis, raloxifene

## Abstract

Estrogen receptor (ER) α is a central regulator of osteoclasts in osteoporosis induced by estrogen deficiency. ERα is regulated through interactions with various coactivators; however, the precise mechanisms of these interactions are not yet fully understood. We screened for proteins that bind to ERα using LC–MS/MS and identified a physical interaction between HSD17B7 and ERα, specifically ERα binding to the 119–172 domain of HSD17B7. This interaction blocked ubiquitin–proteasomal degradation of ERα and increased ERE activity. Estrogen‐deficient mice lacking HSD17B7 in their preosteoclasts showed more severe bone loss than control mice. This was attributed to increased mitochondrial biogenesis through the activation of PLD1–mTOR signaling. Additionally, in preosteoclasts derived from patients with severe osteoporosis, the expression of HSD17B7 and ERα was significantly reduced compared to the control subjects. Finally, raloxifene, which boosts ERα, did not inhibit bone loss without HSD17B7, confirming the modulation of ERα through HSD17B7. Therefore, HSD17B7 regulation is a novel therapeutic approach for alleviating estrogen‐deficient osteoporosis.

## Graphical abstract

In this study, we identified a previously unrecognized function of HSD17B7 as an ERα‐binding protein that serves as a coactivator to influence trabecular bone mass in estrogen deficiency. Specifically, deleting HSD17B7 in myeloid cells enhanced the number of osteoclasts and decreased trabecular bone mass post‐OVX, compared with control OVX, mirroring observations in myeloid ERα KO mice. During increased bone resorption, HSD17B7 expression levels paralleled those of ERα in preosteoclasts. However, HSD17B7 ablation accelerated bone resorption, as evidenced by elevated mitochondrial content and oxidative capacity in preosteoclasts. A transcriptome analysis combined with physiological data pinpointed PLD1 as the target through which HSD17B7 modulates mitochondrial OxPhos, aligning with previous research that highlighted the crucial role of the PLD1 and mTOR signaling pathway in metabolism. An analysis of human subjects revealed that preosteoclasts from severely osteoporotic patients exhibited lower expression levels of HSD17B7 and ERα than those from controls, suggesting that HSD17B7 acts as a positive regulator in human osteoporosis. Lastly, we demonstrated that the selective ER modulator raloxifene had no therapeutic effect on estrogen deficiency‐induced osteoporosis in cKO mice. Therefore, raloxifene's inhibitory effect on osteoclast activation is mediated through HSD17B7, which emphasizes the importance of the HSD17B7–ERα regulatory mechanism in bone homeostasis.

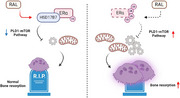

## 1 Introduction

Osteoporosis is a prevalent bone disease marked by decreased bone mineral density (BMD) and the deterioration of bone microarchitecture, which significantly increases the risk of fractures, especially among postmenopausal and elderly people [1]. This condition arises from the uncoupling of bone formation and maintenance, driven by osteoblasts and osteocytes, and bone resorption, driven by osteoclasts, during bone remodeling. In postmenopausal women, the level of the sex hormone estrogen decreases rapidly, accelerating bone resorption by activating osteoclasts [2]. Aging also causes gradual bone loss due to changes in intrinsic factors of the bone (e.g., energy metabolism, oxidative stress, DNA damage, and osteocyte senescence) and external factors (e.g., the ovary and immune system) [3, 4, 5]. Aging and declines in sex hormones thus interact to produce an overall bone phenotype in the elderly that leads to osteoporosis.

The estrogen–estrogen receptor (ER) complex plays a significant role in bone remodeling through several mechanisms. It not only suppresses osteoclast numbers by regulating the apoptosis and cellular metabolism of osteoclast precursors [6, 7], but it also inhibits osteoclastogenesis by regulating the macrophage colony‐stimulating factor (M‐CSF), receptor activator of NF‐κB ligand (RANKL), and osteoprotegerin (OPG) [8, 9, 10]. Thus, estrogen supplementation is theoretically a treatment option for osteoporosis; however, the use of estrogen alone can increase the risk of endometrial, ovarian, and breast cancers [2, 11]. Therefore, selective estrogen receptor modulators (SERMs) such as raloxifene, tamoxifen, and bazedoxifene are used in clinical practice as a form of estrogen replacement therapy [12]. These modulators, bound with coactivators, have estrogen‐like activity in bone and anticancer effects [13]. Unliganded ERα also plays a major positive role in bone formation through the mechanical stimulation of osteoblasts [14, 15, 16], and it has been reported to enhance estrogen response element (ERE) activity in the bone marrow of ovariectomized (OVX) mice [17]. However, the precise mechanisms of action that regulate the ERα complex bound to a SERM and the unliganded ERα complex are still not fully understood.

For this work, we used LC–MS/MS to screen for proteins that bind unliganded ERα in preosteoclasts from OVX mice and found that 17β‐hydroxysteroid dehydrogenase type 7 (HSD17B7) binds unliganded ERα. HSD17B7 catalyzes the conversion of low‐activity keto‐steroid sex hormones into their high‐activity hydroxylated forms, regulating estrogen and cholesterol synthesis [18, 19]. More recently, it has been reported that HSD17B7 regulates cellular energy metabolism [20]. In this study, we demonstrated that myeloid‐specific HSD17B7 knockout mice (cKO) exhibited severe bone loss when estrogen deficiency‐induced osteoporosis was induced. We confirmed that bone loss in these cKOs was due to increased energy metabolism through PLD1–mTOR pathway activity in preosteoclasts. Notably, the ineffectiveness of the raloxifene in treating estrogen‐deficiency osteoporosis in cKO suggests that HSD17B7 is a key regulator in the SERM's action of modulating ERα.

## 2 Results

### 2.1 Preosteoclast ERα Regulation Was Associated With HSD17B7 From OVX Mice

**FIGURE 1:**
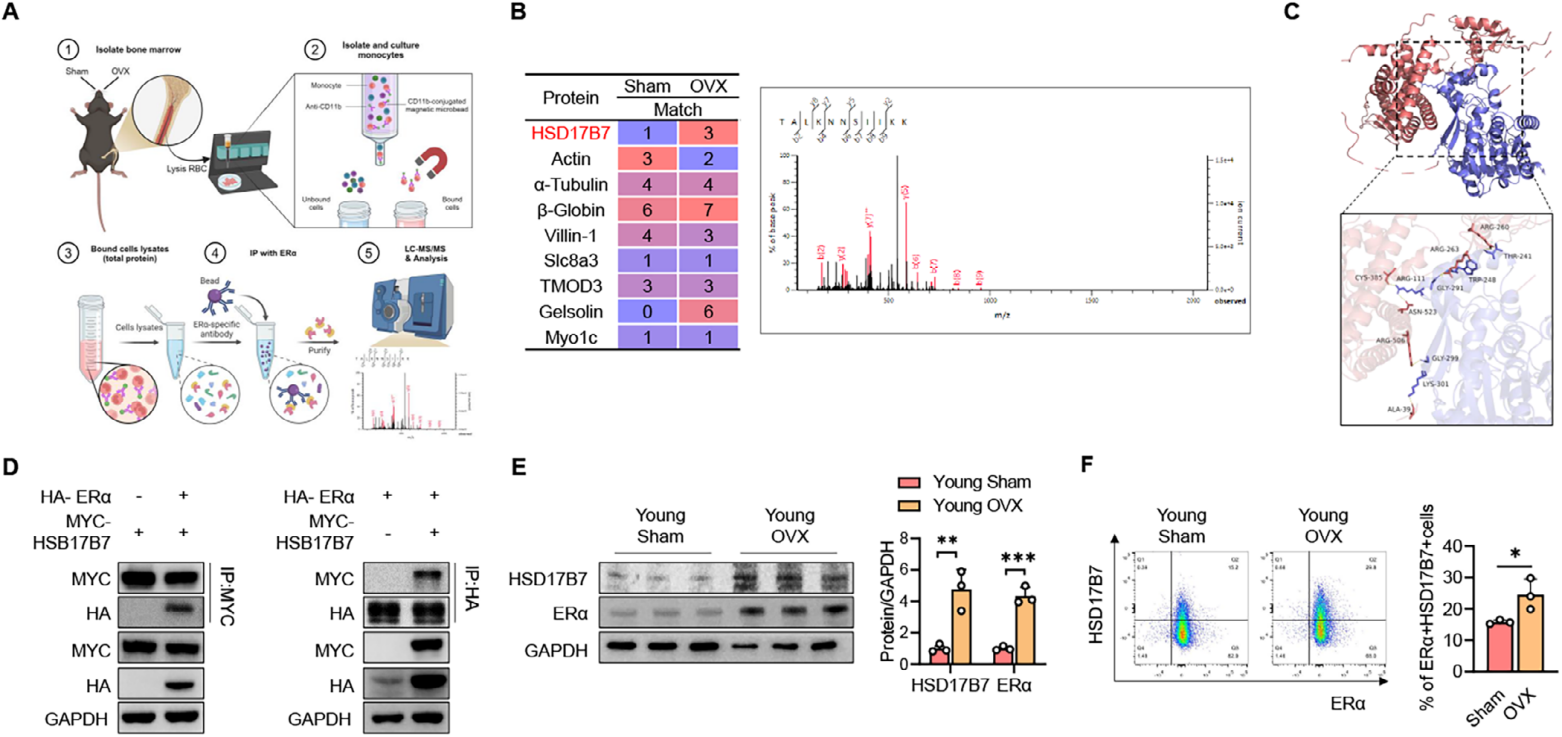
HSD17B as a binding partner with ERα in preosteoclasts. (A) Primary mouse CD11b^+^ bone marrow cell (BMC) proteins were pulled down by biotin‐labeled ERα followed by proteomics. (B) MS/MS spectra of HSD17B7 peptides bound with ERα are shown. (C) The predicted docking modules of HSD17B7 (blue) and ERα (red) were analyzed by AutoDock and exhibited with PyMol. The docking score was 307 and confidence score 0.9585, indicating a strong potential for real binding. (D) After transfecting HEK293T cells with HA‐ERα or MYC‐HSD17B7, a coimmunoprecipitation assay was performed to determine the physical interaction between HSD17B7 and ERα. (E) Western blot analysis of the protein levels of HSD17B7 and ERα in BMCs from Sham/OVX from young mice; *n* = 3. (F) Proportions of HSD17B7 and ERα in CD11b^+^ BMCs from Sham/OVX from young mice. Values presented are the mean ± SEM. *p < 0.05, **p < 0.01, and ***p < 0.001. Figure 1A was created with Biorender.com.

Based on the increase in ERE activity in bone marrow cells (BMCs) following OVX in young mice [17], we used immunoprecipitation with an ERα antibody to verify which proteins interacted with unliganded ERα in myeloid cells 6 weeks after OVX. Next, the proteins bound to ERα were placed on a gel, and the peptides and proteins were identified and estimated by mass spectrometry and the Mascot program (Figure 1A). The data suggest that HSD17B7 can specifically bind to unliganded ERα in the OVX state (Figure 1B), a notion supported by our subsequent predicted docking module analysis (Figure 1C). To explore that possibility, we performed coimmunoprecipitation assays after inserting the ERα and HSD17B7 plasmids. Those results confirmed a physical interaction between HSD17B7 and ERα (Figure 1D). Because ERα is closely related to both aging and estrogen deficiency [21], we checked the expression of ERα and HSD17B7 in CD11b^+^ BMCs. CD11b^+^ BMCs from young OVX mice exhibited a significant increase in ERα expression, compared with young Sham mice. Interestingly, the expression pattern of HSD17B7 was consistent with that of ERα (Figure 1E). To reconfirm the expression patterns of ERα and HSD17B7 in CD11b^+^ BMCs in OVX model, we extracted BMCs from the mice and performed flow cytometry. Those results show that the percentage change in the coexpression of ERα and HSD17B7 in CD11b^+^ BMCs was consistent with the change in protein level (Figures 1F and S1). These results suggest that HSD17B7 might act as a coactivator that binds to ERα and regulates its stability in CD11b^+^ BMCs in mice.

### 2.2 HSD17B7 Interacts With ERα and Stabilizes Its Protein Expression

**FIGURE 2:**
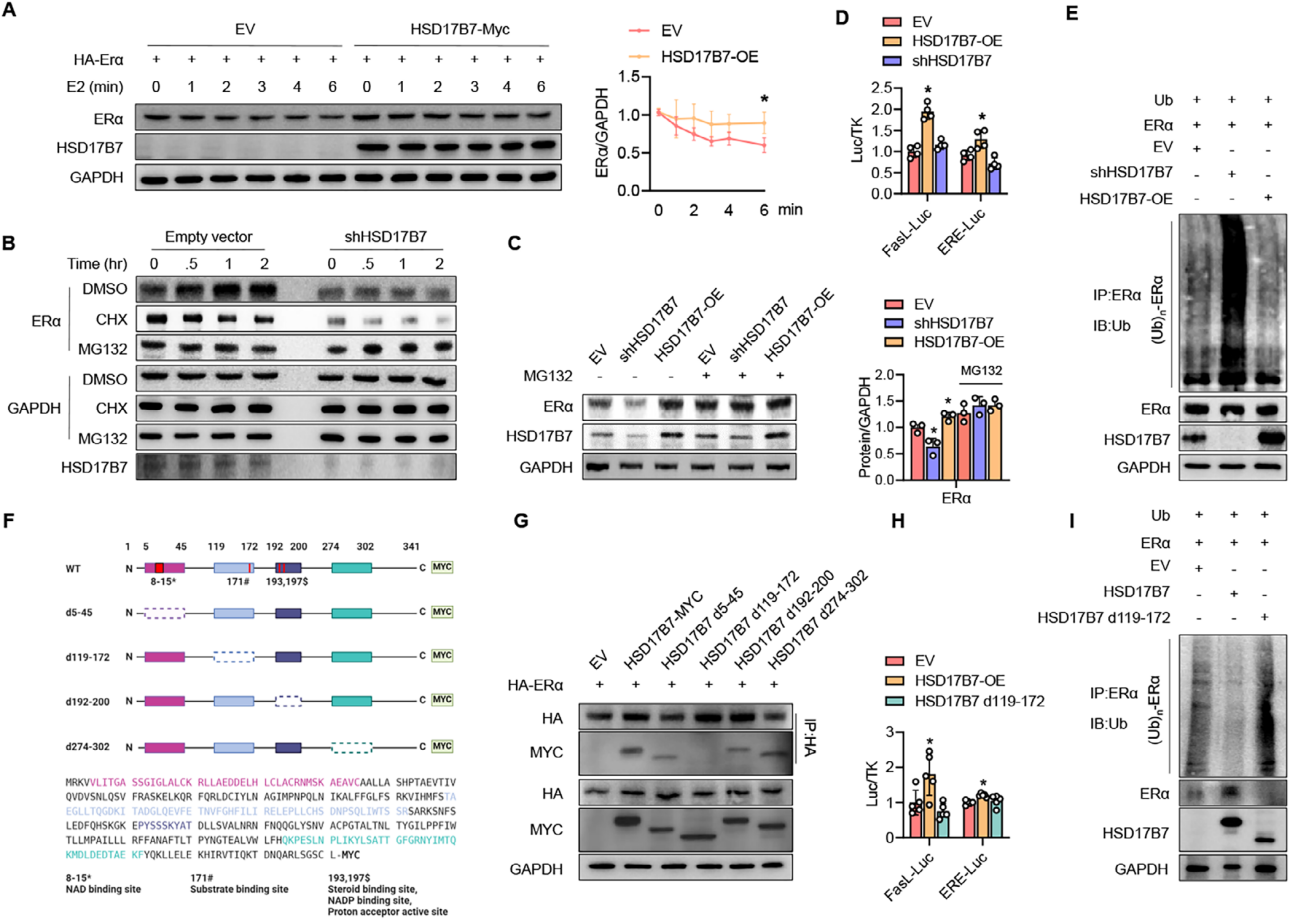
Blocking the ubiquitin–proteasomal degradation of ERα by HSD17B7. (A) HEK293T cells transfected with ERα were treated with estrogen (E2, 10 nM) for the indicated times, and the relative protein levels of ERα were compared (*n* = 3). The blot shown was a representative blot. (B) HEK293T cells were transfected with empty vector or shRNA for HSD17B7. Twenty‐four hours after transfection, the cells were treated with 20 µg/mL cycloheximide (CHX) or 20 µM MG132 for 0.5–2 h. The cell lysates were blotted with GAPDH antibody. The blot shown was a representative blot. (C) HEK293T cells were transfected with ERα and HSD17B7‐OE or shHSD17B7 and then treated with MG132 (20 µM) for 2 h. The cell lysates were immunoblotted with ERα and HSD17B7antibodies, and the results were quantified (*n* = 3). The blot shown was a representative blot. (D) HEK293T cells were cotransfected with HSD17B7‐OE or shHSD17B7, and the FasL and ERE luciferase activity in the cell lysates was measured (*n* = 4). (E) HEK293T cells were cotransfected with Ub, ERα, and HSD17B7‐OE or shHSD17B7. After 24 h, the cells were treated with E2 (10 nM) for 40 min in the presence of MG132 (2 µM), and total cell lysates were immunoprecipitated with anti‐ERα antibodies and immunoblotted with antiubiquitin antibodies. (F–I) Based on the docking prediction results for HSD17B7 and ERα, potential docking sites were deleted from the HSD17B7‐MYC plasmid. (F) Schemes for deleting N‐terminal sequences of HSD17B7. (G) HEK293T cells were cotransfected with HA‐ERα and WT or deletion‐mutant HSD17B7. Coimmunoprecipitation was performed using anti‐HA or anti‐MYC antibodies, and immunoblots were visualized for HSD17B7 and ERα. (H) HEK293T cells were transfected with WT or deletion‐mutant HSD17B7, and FasL and ERE luciferase activities were measured (*n* = 5). (I) HEK293T cells were cotransfected with Ub, ERα, and WT or deletion‐mutant HSD17B7 for 24 h. After 24 h, the cells were treated with E2 (10 nM) for 40 min in the presence of MG132 (2 µM), and total cell lysates were immunoprecipitated using anti‐ERα antibody and immunoblotted using anti‐Ub antibody. Values presented are the mean ± SEM. *p < 0.05 versus EV.

We next investigated the molecular mechanisms by which HSD17B7 regulates ERα. Plasmid a ERα and control vector or HSD17B7 was transfected into HEK293T cells, which were then stimulated with estrogen. In the control group, the protein level of ERα gradually decreased, but in cells overexpressing HSD17B7 under each test condition, the decrease rate was slower (Figure 2A). Furthermore, in HEK293T cells with HSD17B7 knockdown, treatment with cycloheximide led to the rapid degradation of ERα, compared with the empty vector control (Figures 2B and S2), indicating that HSD17B7 increases the stability of ERα in both liganded and unliganded degradation pathways. To further explore the role of HSD17B7 in stabilizing ERα, we evaluated ERα proteasomal degradation. When cells were treated with MG132, a potent proteasome inhibitor, the effect of the HSD17B7‐mediated ERα protein expression change was abolished (Figure 2C), suggesting that HSD17B7‐mediated ERα expression depends on proteasomal protein degradation. Immunoprecipitation was then performed on total protein lysates using ERα antibodies, followed by immunoblotting with ubiquitin antibodies. Those results show that the overexpression of HSD17B7 reduced the ubiquitination of ERα, and HSD17B7 knockdown increased ERα ubiquitination (Figure 2E). Additionally, luciferase assays using FasL‐Luc, which is an ERα target gene [22], and ERE‐Luc confirmed that HSD17B7 overexpression significantly increased ERα transcriptional activity, whereas shHSD17B7 overexpression did not (Figure 2D). Next, we constructed HSD17b7 deletion mutants based on the results of our docking module analysis (Figure S3) and the known functional sites of HSD17B7 [20] to determine where ERα binds to HSD17B7 (Figure 2F). Immunoprecipitation experiments revealed that, compared with the other HSD17B7 deletion mutants, HSD17B7 d119‐172 had a weaker binding affinity with ERα (Figure 2G), and the ubiquitination inhibition effect of HSD17B7 on ERα was not observed when the 119–172 region of HSD17b7 was deleted (Figure 2I). Furthermore, FasL‐Luc and ERE‐Luc confirmed that deleting amino acids 119–172 of HSD17B7 abolished ERα transcriptional activity (Figure 2H). These results indicate that ERα binds to the 119–172 region of HSD17B7, thereby inhibiting the ubiquitin–proteasomal degradation of ERα and increasing its target transcriptional activity. To gain a deeper mechanistic insight into ERα stabilization by HSD17B7, we examined whether ubiquitin‐specific protease 7 (USP7), a specific deubiquitinase (DUB) well known to stabilize ERα [23], and carboxyl terminus of Hsc70‐interacting protein (CHIP), an E3 ubiquitin ligase that ubiquinates unliganded ERα [24], were altered in their binding to ERα by HSD17B7. Interestingly, USP7 bound to ERα without significant changes regardless of HSD17B7 overexpression or knockdown, but CHIP showed decreased binding to ERα upon HSD17B7 overexpression and increased binding to ERα upon HSD17B7 knockdown (Figure S4). These results suggest that HSD17B7 may bind to ERα and sterically hinder the binding of CHIP, an E3 ubiquitin ligase, to unliganded ERα, thereby inhibiting its ubiquitin–proteosomal degradation.

### 2.3 HSD17B7 Deficiency Exacerbates Bone Defects Mediated by Osteoclasts Rather Than Osteoblasts in OVX‐Induced Bone Loss

**FIGURE 3:**
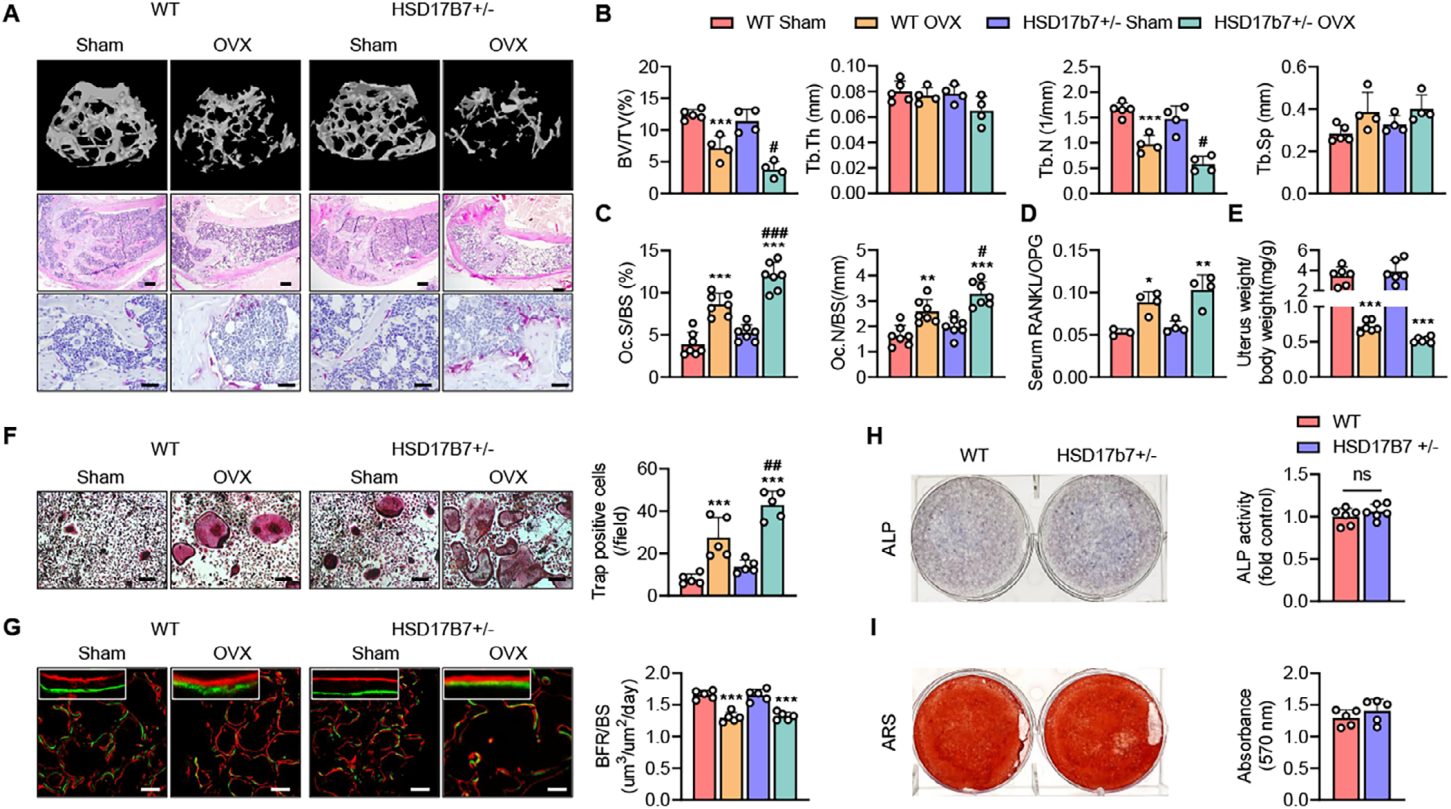
Deficiency of HSD17B7 induces estrogen‐deficiency osteoporosis by increasing the number of osteoclasts without affecting osteoblast function. (A) Representative micro‐CT reconstruction of cancellous bone, H&E staining (scale bars = 250 µm) and TRAP staining (scale bars = 40 µm) images of distal femoral metaphyses (*n* = 4–5 animals/group). (B) Cancellous bone volume (BV/TV, %), trabecular thickness (Tb.Th), trabecular number (Tb.N), and trabecular separation (Tb.Sp) were determined by a micro‐CT analysis. (C) Osteoclast numbers were analyzed in cancellous bone from the distal femur (*n* = 7). (D) The RANKL/OPG ratio in serum was determined (*n* = 3–4). (E) Six weeks after surgery, uterus weight was determined (*n* = 6). (F) Representative TRAP staining images after osteoclastogenesis induction. Scale bars = 40 µm. TRAP‐positive multinucleate cells with three or more nuclei were counted as osteoclasts, and they were scored per field (*n* = 5). (G) Calcein–Alizarin red label staining image and quantitative analysis of BFR (scale bars = 100 µm). (H, I) Representative images of ARS and ALP staining and quantitative analysis (*n* = 4–5). The values presented are the mean ± SEM. *p < 0.05, **p < 0.01, ***p < 0.001 versus WT Sham, ^#^ *p* < 0.05 and ^##^ *p* < 0.01, and ^###^ *p* < 0.001 versus WT OVX.

To investigate the physiological role of HSD17B7 in bone, we generated mice deficient in HSD17B7 in their whole bodies. Because HSD17B7 homozygotes die at embryonic age 10.5 days [25], we used HSD17B7 heterozygotes (HSD17B7^+/−^) and then performed bilateral OVX on both WT and HSD17B7^+/−^ mice. Histological and CT analyses of the distal femur revealed that HSD17B7^+/−^ mice and littermate wild‐type (WT) mice had similar bone masses at 3 months of age (Figure 3A,B). However, after ovariectomy, HSD17B7^+/−^ mice had significantly greater cancellous bone loss than WT mice. Consistently, the trabecular bone volume fraction and trabecular number were significantly lower in the HSD17B7^+/−^ mice than in the WT mice (Figure 3A,B). To further investigate whether bone loss was due to an increase in osteoclast numbers, we performed TRAP staining on the cancellous bone of the distal femur. Results showed that HSD17B7 deficiency significantly increased osteoclast numbers compared to WT mice (Figure 3A,C). Consistently, in vitro osteoclastogenesis induction showed the same trend (Figure 3F). Meanwhile, a significant reduction in uterine weight and an increase in RANKL/OPG ratio confirmed the success of OVX surgery. Still, we found no difference between the genotypes that underwent OVX, indicating no difference in estrogen levels or cytokines for osteoclastogenesis (Figures 3D,E and S5A). To more clearly exclude indirect effects on osteoclastogenesis via the osteogenic lineage, we isolated primary bone marrow stromal cells (BMSCs) from WT and HSD17B7^+/−^ mice and performed osteogenesis. We examined the expression and secretion of osteoclastogenic cytokines. There was no significant difference in the intracellular expression (Figure S5B) and supernatant (Figure S5C) of RANKL and OPG, osteoclastogenic cytokines secreted by the bone‐forming lineage, between WT and HSD17B7^+/−^. Additionally, we treated WT CD11b^+^ cells with conditioned media obtained from the osteogenic differentiation of WT or HSD17B7^+/−^ BMSCs and compared the resulting osteoclastogenesis. The degree of osteoclast differentiation of WT CD11b^+^ cells treated with each conditioned media did not show significant differences among groups (Figure S5D), suggesting that HSD17B7‐deficient osteoblasts do not indirectly affect osteoclast differentiation.

To investigate the effect of HSD17B7 on osteogenesis capacity, Calcein–Alizarin red dual labeling was performed on both genotypes that underwent OVX. The bone‐forming rate results confirmed that HSD17B7^+/−^ mice and their WT littermates had similar osteogenic capacity (Figure 3G). Primary femoral osteoblasts from HSD17B7^+/−^ mice were cultured under osteogenic conditions and did not show differences in osteoblast differentiation or mineralization (Figure 3H,I). Compared with WT mice, primary osteoblasts from HSD17B7^+/−^ mice exhibited significantly reduced HSD17B7 expression. However, the expression levels of other osteoblastic markers (*Col1a1*, *Bglap*, *Runx2*, *Spp1*, and *Osx*) were unaffected by the HSD17B7 deficiency in HSD17B7^+/−^ mice (Figure S5E). Micro‐CT analyses of cortical bone at the mid‐shaft femur showed that tissue mineral density was significantly lower following OVX treatment (Figure S5F,G). However, OVX‐induced cortical bone loss showed almost no difference between the HSD17B7^+/−^ and WT mice (Figure S5F,G), confirming that the loss of HSD17B7 in bone selectively affects trabecular bone during OVX‐induced bone loss.

### 2.4 OVX‐Induced Cancellous Bone Loss Is Aggravated by HSD17B7 Deficiency in Preosteoclasts

**FIGURE 4:**
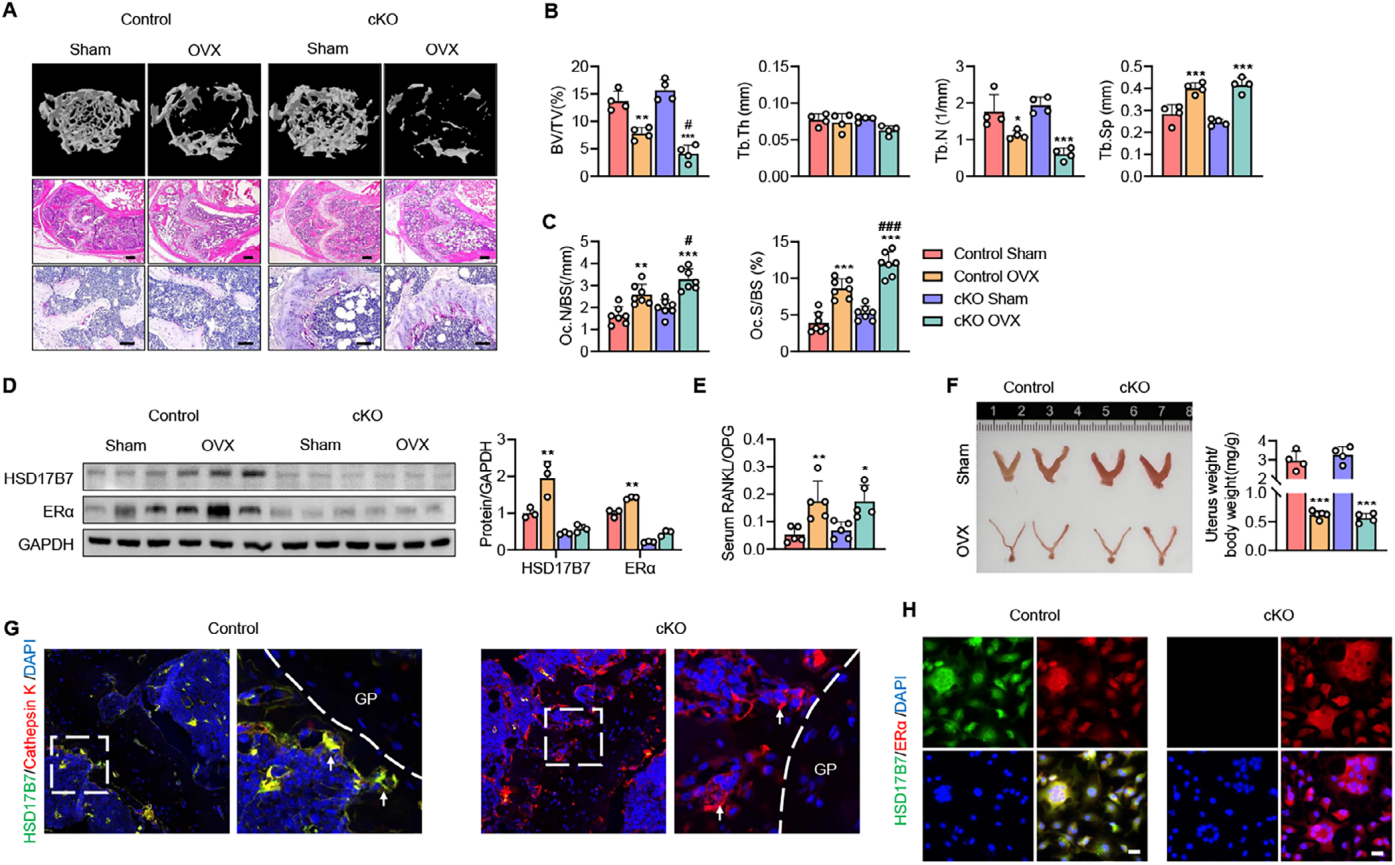
Aggravation of OVX‐induced cancellous bone loss caused by HSD17B7 deficiency in preosteoclasts. (A) Representative micro‐CT reconstruction of cancellous bone, H&E staining (scale bars = 250 µm) and TRAP staining (scale bars = 40 µm) images of distal femoral metaphyses (*n* = 4–5 animals/group). (B) Cancellous bone volume (BV/TV, %), trabecular thickness (Tb.Th), trabecular number (Tb.N), and trabecular separation (Tb.Sp) were determined by a micro‐CT analysis. (C) Osteoclast numbers were analyzed in cancellous bone from the distal femur (*n* = 7). (D) Western blot analysis of the protein levels of HSD17B7 and ERα in Sham/OVX mouse BMCs (*n* = 3). (E) The RANKL/OPG ratio in serum was determined (*n* = 5). (F) Six weeks after surgery, uterus weight was determined (*n* = 4). (G) Representative images of immunofluorescence staining of HSD17B7 (green) and cathepsin K (red) in distal femoral metaphyses. (H) Representative images of immunofluorescence staining of HSD17B7 (green) and ERα (red) in CD11b^+^ osteoclast cells (scale bars = 20 µm). The values presented are the mean ± SEM. *p < 0.05, **p < 0.01, ***p < 0.001 versus Control Sham, ^#^ *p* < 0.05 and ^###^ *p* < 0.001 versus Control OVX.

To further investigate the role of HSD17B7 in preosteoclasts, we crossed *HSD17B7^fl/fl^* mice with *LysM‐Cre* mice to generate myeloid‐specific HSD17B7 knockout (cKO) mice (Figure S6A). A Specific knockout of HSD17B7 in bone marrow was confirmed by Western blot analysis (Figure S6B). cKO mice exhibited a normal phenotype at birth and, at 3 months of age, had bone mass similar to that of *HSD17B7^fl/fl^* littermate mice (Figure 4A). We performed OVX on 3‐month‐old cKO and control mice and sacrificed them 6 weeks postsurgery. As expected, OVX resulted in greater bone loss in cKO mice than in control mice, and the mice treated with a sham operation showed no difference in bone mass (Figure 4A). Consistently, microstructural evaluation of the trabecular bone reflected that the lack of myeloid cell‐specific HSD17B7 exacerbated OVX‐induced trabecular bone loss (Figure 4B). In the OVX state, the number of osteoclasts was significantly higher in cKO mice than in control mice (Figure 4A,C). Additionally, the protective mechanism that increased the expression of ERα and HSD17B7 in BMCs after OVX in control mice was not observed in the cKO OVX mice (Figure 4D). As in the HSD17B7^+/−^mice, the genotypes did not show differences in estrogen levels or cytokines for osteoclastogenesis after OVX (Figure 4E,F). To further investigate the localization of HSD17B7 in the bone marrow, we performed immunofluorescence staining of the trabecular lesion. We confirmed that HSD17B7 coexpressed with the osteoclast marker cathepsin K, supporting its importance in osteoclasts (Figure 4G). In vitro studies corroborated these results. In CD11b^+^ BMCs cultured with M‐CSF and RANKL, HSD17B7 and ERα were coexpressed (Figure 4H), suggesting that HSD17B7 plays a crucial role in trabecular bone resorption by bone marrow osteoclasts. However, this inhibitory effect of HSD17B7 on the number of osteoclasts did not affect the osteoclast differentiation signaling pathway mediated by M‐CSF and RANKL (Figure S6C).

### 2.5 HSD17B7 Ablation Increases Preosteoclast Mitochondrial Content and Oxidative Phosphorylation Capacity

**FIGURE 5:**
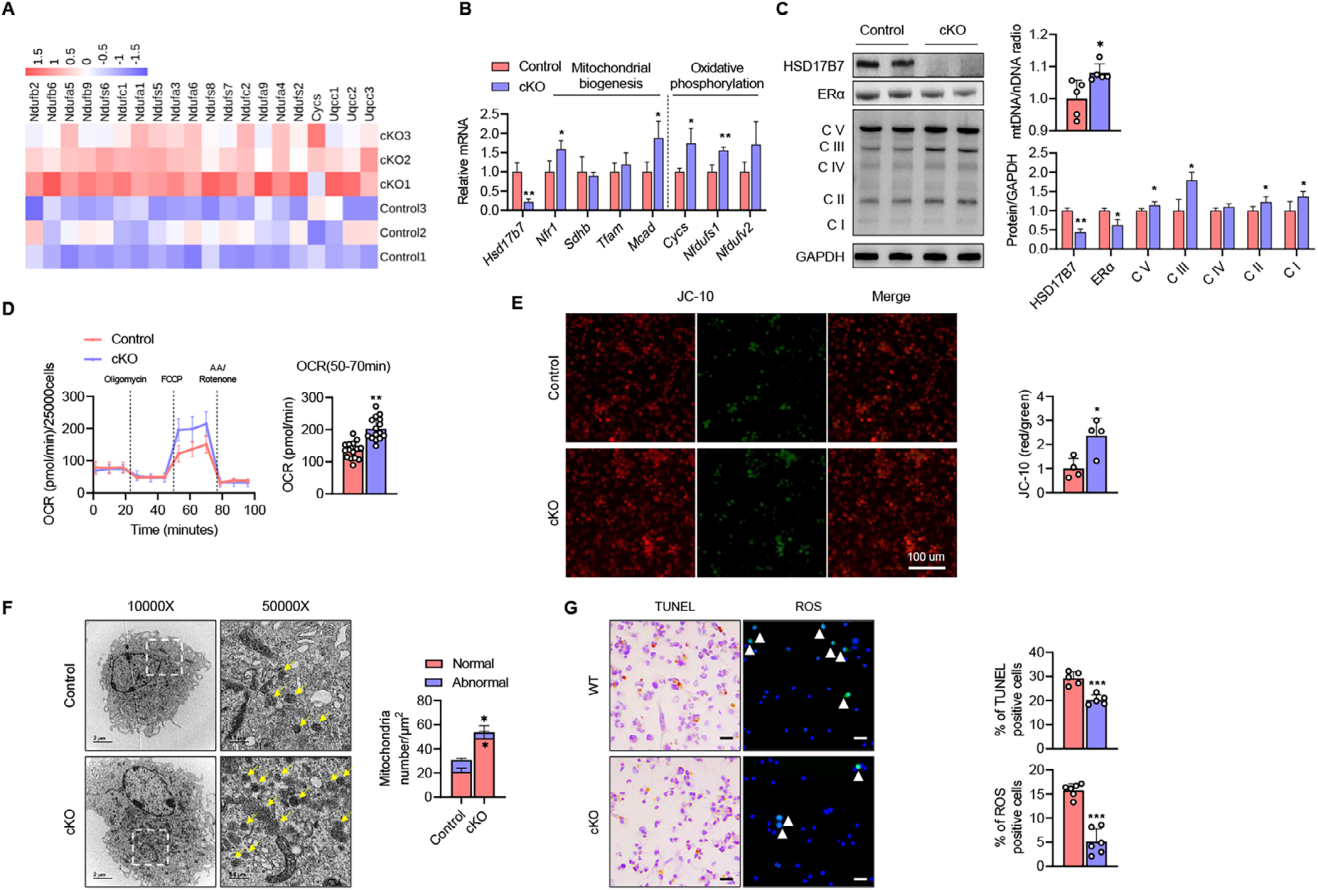
Enhanced mitochondrial function in HSD17B7‐deficient preosteoclasts. (A) Heatmap of the RNA‐seq analysis of mitochondrial complex genes in CD11b^+^ Control/cKO BMCs treated with M‐CSF (10 ng/mL) and RANKL (30 ng/mL). (B) qPCR for marker genes in CD11b^+^ Control/cKO BMCs treated with M‐CSF (10 ng/mL) and RANKL (30 ng/mL; *n* = 4). (C) Representative Western blot results for the OxPhos complex, quantify the results (*n* = 4), and quantitative qPCR analysis of mitochondrial DNA (mtDNA), using nuclear DNA (nDNA) as the standard (*n* = 5). (D) OCR curves in CD11b^+^ Control/cKO BMCs treated with M‐CSF (10 ng/mL), RANKL (30 ng/mL), oligomycin, FCCP, and rotenone/antimycin A (*n* = 5). (E) CD11b^+^ BMCs were stained using JC‐10 and observed via confocal microscopy. Representative images show merged polymeric (red) and monomeric (green) JC‐10 signals. The ratio of polymeric to monomeric JC‐10 was calculated. (F) Representative transmission electron micrographs of M‐CSF (10 ng/mL) and RANKL (30 ng/mL)‐treated CD11b^+^ Control/cKO BMCs and quantitative analysis showing the numbers of mitochondria with normal and abnormal morphology. (G) Fragmentation of cellular DNA was detected by a TUNEL assay. Intracellular reactive oxygen species (ROS) were detected by H2DCFDA (green) (scale bars = 40 µm). The values presented are the mean ± SEM. *p < 0.05, **p < 0.01, and ***p < 0.001.

To elucidate the mechanism by which HSD17B7 regulates preosteoclasts, we treated CD11b^+^ BMCs from cKO mice with RANKL for 2 days and then performed an RNA sequencing analysis. Considering that the mitochondrial OxPhos of preosteoclasts plays an essential role in the number and function of osteoclasts via the estrogen–ER complex [7, 26, 27], we focused on the expression of mitochondria‐related genes. The heatmap in Figure 5A displays the increased expression of mitochondrial complex genes in cKO mice, compared with control mice. A qPCR analysis confirmed alterations in mitochondrial biogenesis and OxPhos gene expression (Figure 5B). These results are consistent with the increased mitochondrial DNA content and enhanced expression of oxidative phosphorylation complexes found in preosteoclasts from cKO mice (Figure 5C). To ascertain whether the increased mitochondrial content in cKO preosteoclasts correlates with elevated respiratory function, Seahorse XF mitochondrial stress tests were conducted on differentiated preosteoclasts from CD11b+ BMCs with both genotypes. We observed a significant increase in maximum OCR in cKO cells, indicating that HSD17B7 regulates energy metabolism in preosteoclasts by modulating OxPhos (Figure 5D). The results of measuring mitochondrial membrane potential, an indicator of mitochondrial activity, also showed higher levels in the cKO group than the control group (Figure 5E). Consistently, an electron microscopy analysis of preosteoclasts revealed a higher number of healthier mitochondria in cKO mice than in control mice (Figure 5F). These results were corroborated by in vitro studies. TUNEL‐positive cells and reactive oxygen species (ROS)‐positive cells were significantly reduced in preosteoclasts from cKO mice, compared with control mice, indicating that the apoptotic process within preosteoclasts of cKO mice was inhibited, and oxidative stress levels were decreased (Figure 5G).

### 2.6 HSD17B7 Affects Osteoclast Function Through the PLD1–mTOR Signaling Pathway

**FIGURE 6:**
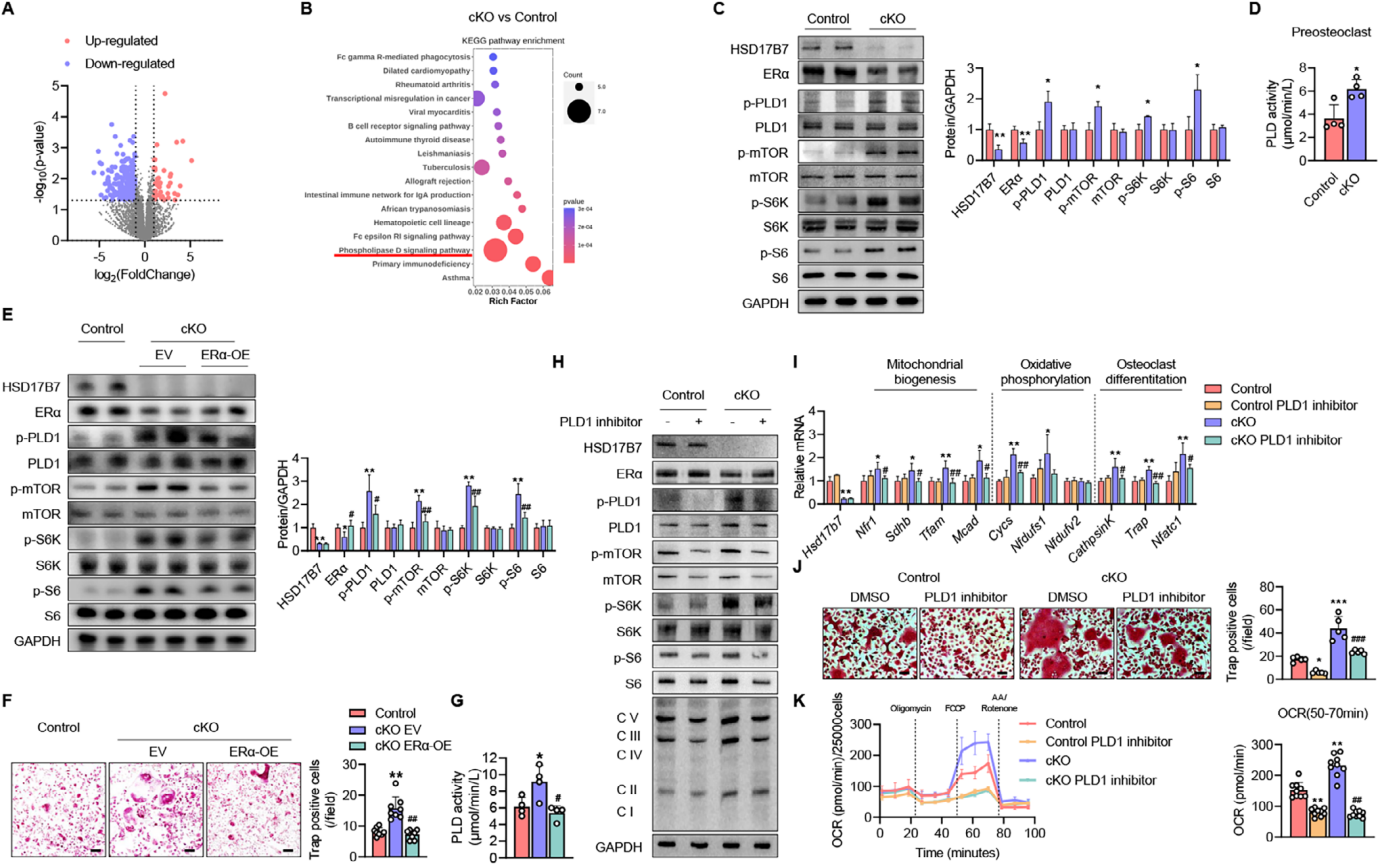
Enhanced oxidative capacity of HSD17B7 cKO mice via the PLD1–mTOR signaling pathway. (A) Volcano plot showing the log2 fold‐difference in M‐CSF (10 ng/mL) and RANKL (30 ng/mL)‐treated CD11b^+^ Control/cKO BMCs, assessed using a StringTie analysis of RNA‐seq data. Red and blue dots represent upregulated and downregulated DEGs, with > twofold change and *p* < 0.05, respectively. Each sample was analyzed in triplicate. (B) Bubble plots show significant changes in the KEGG analysis. (C) Representative Western blot analysis of the PLD1–mTOR signaling pathway and the results were quantified (*n* = 4). (D) PDL1 activity from preosteoclasts. (E–G) CD11b^+^ cKO BMCs were transfected with ERα or empty vector (EV) by electroporation for 24 h. (E) Representative the expression patterns of PLD1–mTOR signaling pathway were determined via Western blot and quantify the results (*n* = 4). (F) Representative TRAP staining after osteoclastogenesis induction. Scale bars = 100 µm. TRAP‐positive multinucleate cells with three or more nuclei were counted as osteoclasts and were scored per field (*n* = 8). (G) PDL1 activity from preosteoclasts. (H–K) CD11b^+^ Control/cKO BMCs were treated with VU0359595 (1 µM) for 24 h. (H) Representative the expression patterns of the PLD1–mTOR signaling pathway and OxPhos complex were determined via Western blotting. (I) qPCR for marker genes after treatment with VU0359595. (J) Representative TRAP staining images after osteoclastogenesis induction. Scale bars = 200 µm. TRAP‐positive multinucleate cells with three or more nuclei were counted as osteoclasts, and they were scored per field (*n* = 5). (K) OCR curves in VU0359595‐treated CD11b^+^ Control/cKO BMCs also treated with oligomycin, FCCP, and rotenone/antimycin A (*n* = 3). The values presented are the mean ± SEM. *p < 0.05, **p < 0.01, ***p < 0.001 versus Control, ^#^ *p* < 0.05, ^##^ *p* < 0.01, ^###^ *p* < 0.001 versus cKO or EV‐treated cKO.

To elucidate the molecular mechanisms by which HSD17B7 regulates osteoclast numbers and energy metabolism, we further analyzed the RNA sequencing data. The volcano plot in Figure 6A shows genes that were upregulated (in red) or downregulated (in blue) in cKO mice, compared with control mice. The KEGG pathway analysis (https://www.genome.jp/kegg/pathway.html) performed on transcriptomes that increased in preosteoclasts from cKO mice revealed significant alterations in the PLD1 signaling pathway (Figure 6B). Because PLD1 and mTOR have been reported to have important roles in osteoclast differentiation and energy metabolism [28, 29, 30], we investigated the PLD1–mTOR pathway. We found that proteins related to the PLD1 pathway and PLD activity were consistently and significantly increased in cKO mice, compared with the control group (Figure 6C,D). To confirm whether the increase in the PLD1–mTOR signaling pathway caused by HSD17B7 deficiency in preosteoclasts was due to ERα, which was previously identified as being regulated by HSD17B7, we overexpressed ERα in HSD17B7 knockout preosteoclasts and examined the PLD1–mTOR signaling pathway. Overexpression of ERα in preosteoclasts of cKO mice suppressed the PLD1–mTOR pathway (Figure 6E) and significantly reduced PLD activity (Figure 6G) compared to the empty vector‐treated cKO preosteoclast. Additionally, the number of osteoclasts was decreased in the overexpressed ERα of cKO (Figure 6F). These results indicated that the PLD1–mTOR pathway, regulated by HSD17B7, operates in an ERα‐dependent manner. To determine whether PLD1 signaling affects osteoclast function and cell metabolism, we treated cKO preosteoclasts with VU0359595, a PLD1 inhibitor, and examined cell metabolic status and osteoclast number. The PLD1 inhibitor effectively suppressed PLD1–mTOR signaling in cKO preosteoclasts (Figures 6H and S7). Under these conditions, the PLD1 inhibitor reduced the protein expression of OxPhos complexes (Figure 6H), the expression of transcripts related to mitochondrial biogenesis and oxidative phosphorylation (Figure 6I), and oxygen consumption rate (OCR; Figure 6K) in cKO preosteoclasts compared to DMSO‐treated cKO preosteoclasts. Additionally, the PLD1 inhibitor partially suppressed the increased expression of mRNA related to osteoclast differentiation markers (Figure 6I) and the increased number of osteoclasts (Figure 6J) in cKO mice during the osteoclast differentiation process. These results suggested that the increased osteoclast metabolism and activity observed in HSD17B7 deficiency are mediated through the PLD1 pathway.

### 2.7 HSD17B7–ERα Expression Is Attenuated in Patients With Severe Osteoporosis

**FIGURE 7:**
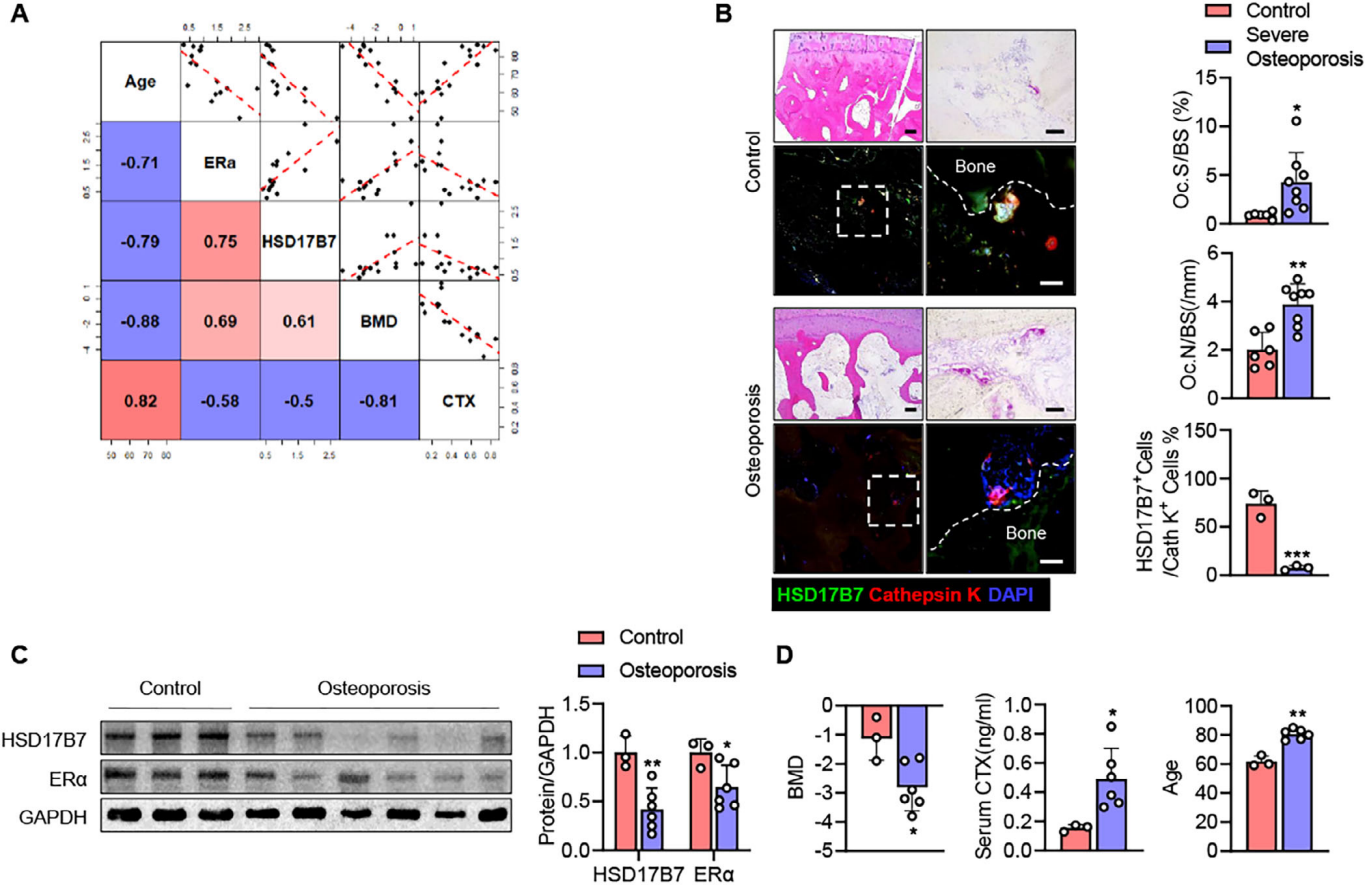
Attenuation of HSD17B7–ERα expression in severely osteoporotic patients. Human CD11b^+^ BMCs were treated with M‐CSF (25 ng/mL) and RANKL (50 ng/mL) for 7 days. (A) Scatterplots of HSD17B7 expression and various bone parameters. The coefficient of determination was used to compare the association between HSD17B7 expression and the bone parameters, with red and blue representing negative and positive correlation, respectively (*n* = 16). (B) Representative H&E staining (scale bars = 250 µm), TRAP staining (scale bars = 40 µm) and immunofluorescence staining (scale bars = 40 µm) images of osteoclasts in control subjects and osteoporotic patients. (C) Human CD11b^+^ BMCs were treated with M‐CSF and RANKL for 7 days, and the expression of HSD17B7 and ERα proteins was analyzed using Western blotting. (D) Clinical parameters of control subject and osteoporotic patients. The values presented are the mean ± SEM. *p < 0.05, **p < 0.01, ***p < 0.001 versus control. Control: T‐score >‐ 1, Osteoporosis: T‐score ← 2.5, Severe osteoporosis: T‐score ← 2.5 with bone fracture.

To determine the clinical significance of our findings, CD11b^+^ BMCs obtained from human subjects were induced to undergo osteoclastogenesis and analyzed for the expression of HSD17B7 and ERα. Similar to the mouse studies, HSD17B7 and ERα in human preosteoclasts decreased with age (Figure 7A). Positive correlations were found between HSD17B7 and ERα expression, HSD17B7 and BMD, and ERα and BMD (Figure 7A), supporting animal studies that showed HSD17B7 enhances both ERα protein levels and BMD. In contrast, both HSD17B7 and ERα exhibited negative correlations with serum type I collagen C‐terminal telopeptide (CTX), a marker of osteoclast activity (Figure 7A). To further examine the expression of HSD17B7 in osteoclasts from patients with osteoporosis, we performed hematoxylin and eosin (H&E) and TRAP staining on femoral heads, revealing that trabeculae were sparser and the number of mature osteoclasts was significantly higher in osteoporosis patients than in the control group. Consistent with the results of our in vivo and ex vivo experiments, HSD17B7 expression was high in osteoclasts from the control group and significantly lower in osteoclasts from patients with severe osteoporosis (Figure 7B). Finally, we confirmed HSD17B7 and ERα expression in preosteoclasts from patients with severe osteoporosis (Figure 7C). Compared with the control group, patients with severe osteoporosis had lower bone density and higher CTX levels, and the expression of HSD17B7 and ERα was significantly lower (Figure 7D). These findings suggest that decreased expression of HSD17B7 and ERα in preosteoclasts is highly associated with osteoporosis in humans.

### 2.8 A Lack of HSD17B7 Abolishes the Osteoclast‐Inhibitory Function of Raloxifene

**FIGURE 8:**
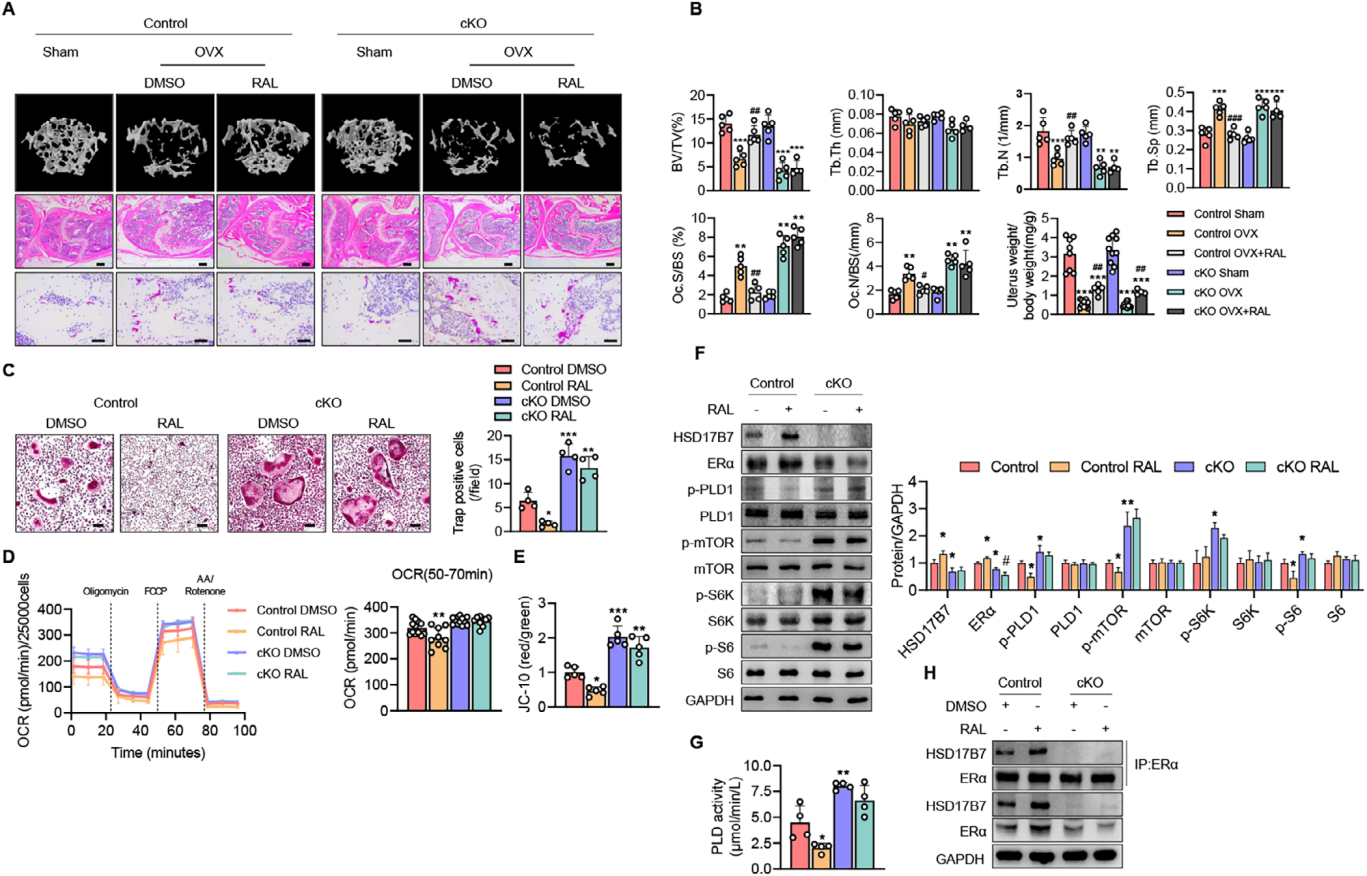
The anti‐bone‐resorption effect of raloxifene in estrogen‐deficient osteoporosis is mediated by HSD17B7. (A) Control and cKO mice were treated with raloxifene (RAL) (0.5 mg/kg) via subcutaneous injection 5 days/week for 5 weeks during the OVX program. Representative micro‐CT reconstruction of cancellous bone, H&E staining (scale bars = 250 µm), and TRAP staining (scale bars = 40 µm) images of distal femoral metaphyses (*n* = 4–5 animals/group). (B) Cancellous bone volume (BV/TV, %), trabecular thickness (Tb.Th), trabecular number (Tb.N), trabecular separation (Tb.Sp), and uterus weight were determined by a micro‐CT analysis. (C) Representative TRAP staining images after osteoclastogenesis induction. Scale bars = 100 µm (*n* = 5). (D) OCR curves from CD11b^+^ Control/cKO BMCs treated with RAL (3 µM), M‐CSF (10 ng/mL), RANKL (30 ng/mL), oligomycin, FCCP, and rotenone/antimycin A (*n* = 3–4). (E) Calculation of the ratio of polymeric to monomeric JC‐10. (F) CD11b^+^ Control/cKO BMCs were treated with RAL (3 µM) for 24 h, and representative the expression patterns of the PLD1–mTOR signaling pathway were determined via Western blotting and the results were quantified (*n* = 3). (G) PDL1 activity from preosteoclasts. (H) Co‐IP and immunoblot analyses of CD11b^+^ Control/cKO BMCs treated with RAL. *p < 0.05, **p < 0.01 versus Control Sham or Control DMSO, ^#^ *p* < 0.05, ^##^ *p* < 0.01, ^###^ *p* < 0.001 versus Control OVX, cKO OVX, or cKO DMSO.

It has been reported that SERMs bind to ER, inhibit osteoclast activity [12], and block breast cancer cell proliferation because they inhibit PLD1 [31, 32]. Interestingly, our present study shows that HSD17B7 stabilizes ERα to activate ERα target genes and inhibits the PLD1–mTOR pathway to block osteoclast activity. In other words, we observed a significant overlap in the roles of HSD17B7 and SERMs. Thus, we hypothesized that HSD17B7 would be related to the effect of SERMs. We treated cKO mice and control mice with a SERM after OVX and observed the bone phenotype. For this experiment, we used AutoDock Vina to simulate molecular docking between HSD17B7 and raloxifene, with a binding energy of −8.5 kcal/mol indicating a significant potential for actual binding (Figure S8). To determine the effect of raloxifene on cKO mice, we subcutaneously injected 8‐week‐old female cKO mice with raloxifene 5 days a week for 5 weeks during the OVX protocol [33, 34]. Histology and CT showed that, following OVX treatment, raloxifene treatment increased trabecular bone in control mice but had minimal effects on cKO mice (Figure 8A). Consistent with those changes, far fewer osteoclasts were formed in the presence of raloxifene in control mice, but raloxifene treatment did not lead to significant changes in the number of osteoclasts in cKO mice either in vivo or in vitro (Figure 8A–C). These results suggest that raloxifene increases bone mass in estrogen‐deficient mice, similar to the effects of estrogen on bone, but it does not reverse bone loss in HSD17B7‐deficient mice. Previous results have demonstrated that 17β‐estradiol inhibits mitochondrial function and promotes apoptosis in preosteoclasts [7]. Raloxifene also regulates mitochondrial‐mediated apoptosis and inhibits mitochondrial and peroxisomal β‐oxidation [35, 36]. Therefore, we performed Seahorse XF mitochondrial stress tests on preosteoclasts to investigate whether raloxifene interacts with HSD17B7 in ways that affect mitochondrial function in preosteoclasts. We observed that raloxifene significantly reduced OCR in control preosteoclasts, but it did not affect cKO preosteoclasts (Figure 8D). Similarly, raloxifene decreased mitochondrial membrane potential in control preosteoclasts but not in cKO preosteoclasts (Figure 8E), suggesting that HSD17B7 might be an essential mediator of the mitochondrial function‐regulating effects of raloxifene in preosteoclasts. Next, we evaluated the expression of the PLD1 pathway in preosteoclasts after raloxifene treatment. Interestingly, RAL‐stimulated control preosteoclasts showed significantly reduced expression of PLD1‐related protein and PLD activity, along with increased expression of HSD17B7 and ERα, compared with untreated control cells. However, that effect was absent in cKO preosteoclasts (Figure 8F,G). Through coimmunoprecipitation studies after raloxifene treatment of control and cKO preosteoclasts, we observed an increase in the physical interaction between ERα and HSD17B7 in control mice but not cKO mice (Figure 8H). These findings suggest that raloxifene exerts its effects via HSD17B7 in preosteoclasts and suppresses bone loss in estrogen‐deficient osteoporosis by regulating the expression of HSD17B7–ERα.

## 3 Discussion

In this study, we identified a previously unknown function of HSD17B7 as a coactivator influencing cancellous bone mass in estrogen deficiency. Specifically, the deletion of HSD17B7 in myeloid cells resulted in increased osteoclast numbers and decreased cancellous bone mass after OVX, compared to control OVX, similar to the results observed in myeloid ERα KO mice [37]. During increased bone resorption, HSD17B7 expression levels showed a pattern similar to that of ERα in preosteoclasts. However, deletion of HSD17B7 further accelerated bone resorption by increasing the mitochondrial content and oxidative capacity of preosteoclasts. Combining transcriptomic analysis with physiological data revealed that the PLD1 pathway is a target pathway of HSD17B7, which regulates mitochondrial oxidative phosphorylation. This finding is consistent with previous studies demonstrating that PLD1 and the mTOR signaling pathway play crucial roles in metabolism [28, 29, 30, 38]. An analysis of human subjects revealed that preosteoclasts from severely osteoporotic patients exhibited lower expression levels of HSD17B7 and ERα than those from controls, suggesting that HSD17B7 acts as a positive regulator in human osteoporosis. Lastly, we demonstrated that the selective ER modulator raloxifene had no therapeutic effect on estrogen deficiency‐induced osteoporosis in cKO mice. Therefore, raloxifene's inhibitory effect on osteoclast activation is mediated through HSD17B7, which emphasizes the importance of the HSD17B7–ERα regulatory mechanism in bone homeostasis.

In this study, we used LC–MS/MS to find that HSD17B7 binds to ERα and reconfirmed that finding by in vitro coimmunoprecipitation. This binding occurred at 119–172 on HSD17B7, which includes the substrate binding site and the docking prediction site, rather than the steroid and NADP binding sites of HSD17B7 [20]. The binding of the two proteins enhanced the stability of ERα by inhibiting its ubiquitin–proteasomal degradation. Evidence from diverse expression patterns supports this mechanism of ERα regulation by HSD17B7. First, when young mice are rendered estrogen‐deficient, a complementary increase in ERα is observed in myeloid cells [17, 21], at which time the expression of HSD17B7 is significantly increased. Second, in aged rodents, ERα expression is markedly reduced by nonligand‐dependent ubiquitination [39], and HSD17B7 is also reduced in the same manner in human preosteoclasts. Third, ERα expression is consistently reduced in HSD17B7 KO preosteoclasts, compared with control cells. Lastly, the expression of HSD17B7 and ERα in patient‐derived preosteoclasts showed a significant positive correlation. Because ERα has various posttranslational modification sites that regulate its ubiquitin–proteasomal degradation [40, 41, 42], binding to HSD17B7 is thought to affect stability by inducing changes in those lysine and serine residues. Another possibility is that HSD17B7 inhibits ERα binding to CHIP, which is an E3 ubiquitin ligase. It has been reported that CHIP promotes the ubiquitin–proteasomal degradation of unliganded ERα in a state of long‐term estrogen depletion associated with aging [24]. In addition, the present study clearly observed the binding of CHIP to ERα in an opposite manner when HSD17B7 was overexpressed and knocked down, suggesting that HSD17B7 may sterically hinder the binding of ERα to CHIP. On the other hand, it is also possible that ERα regulates HSD17B7. ERα bound to the ligand was recruited to the promoter of HSD17B7 and stimulated its expression [19]. Furthermore, we found that the expression of HSD17B7 increased when control preosteoclasts were treated with raloxifene. Thus, the regulatory mechanism of HSD17B7 and ERα is thought to be a feed‐forward loop.

Through a transcriptome analysis, we found that the phospholipase D signaling pathway was a significant mechanism in the increase in the number of osteoclasts in HSD17B7‐deficient preosteoclasts. PLD1 is an upstream factor of the mitogenic activation of mTOR signaling and regulates cell growth through the PLD1–mTOR–S6K1 axis [30]. Consistent with these results, the osteoclastic activity of HSD17B7 knockout preosteoclasts accompanied the activation of the PLD1–mTOR–S6K1 axis, and that osteoclastic activity was suppressed by a PLD1 inhibitor. PLD1 is involved in synoviocyte activation and the expression of various cytokines in rheumatoid arthritis [43], and PLD1 inhibitors have been reported to alleviate the symptoms of collagen‐induced arthritis and inhibit osteoclast activity [28], supporting our findings in this study. Meanwhile, PLD1–mTOR is a central regulator of not only cell growth but also cellular metabolism. PLD1 or mTOR inhibition suppresses oxygen consumption and mitochondrial capacity and increases ROS levels, leading to cell death [44, 45, 46, 47]. HSD17B7 KO preosteoclasts showed increased OCR, enhanced mitochondrial biogenesis, and decreased ROS, which reduced the number of apoptotic cells. All of these metabolic‐related preosteoclast phenotypes were reversed by a PLD1 inhibitor, suggesting that metabolic events associated with HSD17B7 are regulated by PLD1–mTOR signaling. Although we could not provide direct evidence that the phenotypes of HSD17B7 KO preosteoclasts were entirely related to ERα, we can speculate that the effects of HSD17B7 are highly associated with ERα based on the results that PLD1 signaling was reduced in HSD17B7 KO preosteoclasts that overexpress ERα, which leads to decreased cellular metabolism and osteoclast number, the result that raloxifene, which binds to ERα and acts on preosteoclasts, did not improve the bone phenotype in HSD17B7 KO mice and on reports that increased metabolic activity in ERα KO preosteoclasts [7] and the overexpression of PLD1 were observed in ER‐negative breast cancer cells [48, 49].

Raloxifene, a SERM, exploits the positive effects of ERα on bone to alleviate osteoporosis caused by estrogen deficiency. It has been used extensively clinically and has shown various pharmacological properties, including antiosteoporotic, antiviral, immunomodulatory, and anticancer activities [12, 35, 50, 51]. We introduced raloxifene to investigate the role of HSD17B7 in estrogen‐deficiency osteoporosis for three reasons. First, in cKO and control OVX models, the RANKL‐to‐OPG ratio remained the same between the OVX genotypes, and estrogen was reduced by the same amount. However, the degree of bone resorption was different, suggesting that ERα, which is highly related to HSD17B7 in preosteoclasts, might have estrogen‐independent bone protective effects. Second, although the exact mechanism has not yet been fully elucidated, raloxifene is a PLD1 inhibitor [32]. Because HSD17B7 regulated osteoclast activity through the PLD1 signaling pathway, we expected raloxifene to be highly related to HSD17B7. Third, the in silico structure prediction and molecular docking analysis predicted that HSD17B7 and raloxifene would have a high binding affinity. We have demonstrated that raloxifene did not affect bone phenotypes in OVX mice lacking HSD17B7 in their preosteoclasts, indicating that the osteoprotective effect of raloxifene occurs through HSD17B7. The expression of HSD17B7 in preosteoclasts decreased with increasing age, which could explain the clinical decline seen in the effect of raloxifene on BMD with increasing age after menopause [52, 53]. Thus, boosting the protein level of HSD17B7 could be one treatment method for improving the effect of raloxifene on osteoporosis. On the other hand, therapeutic strategies that utilize an agonist that only increases HSD17B7 may be risky. Since HSD17B7 catalyzes the reaction that converts estrone to estradiol, studies have been conducted to inhibit the function of HSD17B7 in estrogen‐dependent breast cancer [19, 54]. Additionally, a recent study reported that HSD17B7 promotes cholesterol formation in macrophages, inducing M1 polarization of these cells in the liver and thereby aggravating nonalcoholic fatty liver disease [55]. Therefore, rather than developing a treatment that increases HSD17B7 activity alone, it is thought that a strategy to establish an estrogen‐independent ERα modulator through coadministration with SERMs and an optimal combination is a more appropriate osteoporosis treatment strategy.

In summary, this study has demonstrated that HSD17B7 upregulates and increases ERα expression in preosteoclasts, inhibiting their metabolic activity and controlling bone resorption. Therefore, HSD17B7 regulation could be a novel therapeutic approach to alleviating osteoporosis in postmenopausal patients.

## 4 Materials and Methods

### 4.1 Animal Experiments

WT C57BL/6J mice were obtained from Damul Science (Daejeon, Korea). HSD17B7^+/−^ mice were obtained from Gempharmatech (Nanjing, China), HSD17B7^fl/fl^ mice were obtained from Cyagen (Santa Clara, CA, USA), and LysM‐Cre mice were obtained from Jackson Laboratory (Bar Harbor, ME, USA). Myeloid‐specific HSD17B7 knockout mice (cKO) mice were generated by mating HSD17B7^fl/fl^ mice with LysM‐Cre mice. HSD17B7^fl/fl^ littermates served as controls. Animals were maintained on a 12‐h light/dark cycle at 23 ± 1°C with free access to food and water. The genotypes of the mice were determined by polymerase chain reaction (PCR) using tail tissues. The primer sequences used in the PCR are listed in Table S1. Eight‐week‐old female mice were subjected to bilateral OVX or sham surgery, and the mice were sacrificed after 6 weeks. The bone parameters of cancellous and cortical bone from the distal femur were analyzed using a SKYSCAN 1076 Micro‐CT unit (SkyScan, Kontich, Belgium) installed in the Center for University‐wide Research Facilities (CURF) at Jeonbuk National University. The study protocol was approved by the Institutional Animal Care and Use Committee of Jeonbuk National University Hospital (Permit No: JBUH‐IACUC‐2020‐20).

### 4.2 In Vitro Osteoclast Differentiation

Femoral CD11b^+^ BMCs from 8‐week‐old mice were used for osteoclastogenesis. Briefly, mouse femoral BMCs were extracted, and the cell suspensions were passed through a 70‐µm mesh filter screen. Red blood cells were removed using ACK lysing buffer. The following day, the supernatant cells were collected and combined with CD11b (Invitrogen, Carlsbad, CA, USA), and antibiotin microbeads (Miltenyi Biotec, San Diego, CA, USA) were used for positive sorting. The isolated cells were seeded at a density of 1 × 10^6^ in six‐well plates and cultured for 2 days with the addition of 30 ng/mL M‐CSF (PeproTech, London, UK). To induce osteoclast formation, M‐CSF (10 ng/mL) and RANKL (30 ng/mL, PeproTech) were added to the culture medium. After 3 days of osteoclast differentiation, tartrate‐resistant acid phosphatase (TRAP) multinucleated cells were detected using a TRAP staining kit (Cosmo Bio Co., Ltd., Tokyo, Japan). The procedures for human osteoclast extraction and osteoclastogenesis were similar to those in a previous study [21]. Briefly, human CD11b^+^ BMCs were extracted, and 25 ng/mL M‐CSF and 50 ng/mL RANKL were added for osteoclast differentiation over 7 days.

### 4.3 LC–MS/MS and Peak Alignment

Immunoprecipitated proteins differentially expressed in CD11b^+^ BMCs from sham and OVX mice were identified by staining SDS gels using colloidal Coomassie blue (Abcam, Cambridge, UK). Proteins were isolated from gel bands and digested with trypsin. Tryptic peptides were analyzed for relevant proteins using a high‐resolution mass spectrometer (LTQ‐Orbitrap Velos, Thermo Fisher Scientific, Waltham, MA, USA) as described previously [56].

### 4.4 Patients

We evaluated patients who underwent hip arthroplasty at Jeonbuk National University Hospital between August 2022 and August 2023. The inclusion and exclusion criteria were described previously [21]. Sixteen patients met the criteria. All cases were reviewed according to the World Health Organization Diagnostic Criteria for Osteoporosis [57] and the American Association of Clinical Endocrinologists staging system [58]. This study was performed with the approval of the institutional review board at Jeonbuk National University Hospital, and the requirement for informed consent was waived (IRB number, JBUH 2023‐12‐023).

### 4.5 Histology

Mouse femurs were fixed in 4% paraformaldehyde, decalcified, and soaked in 10% EDTA for 1 month until the bone softened evenly to complete the decalcification. The femurs were then embedded in paraffin, and cut into 5 µm transverse sections at different levels. The sections were stained with H&E and TRAP after rehydration.

For immunofluorescence staining, the sections were deparaffinized, rehydrated, and subjected to antigen retrieval. After being blocked with 5% BSA (GenDEPOT, Barker, TX, USA) to prevent nonspecific staining, the slides were incubated with primary anti‐HSD17B7 (14854‐1‐AP, ProteinTech Group, Chicago, IL, USA) and anticathepsin K (sc‐48353, Santa Cruz Biochemicals, Dallas, TX, USA) overnight at 4°C. Alexa Fluor 488‐conjugated anti‐rabbit IgG (1:100 Invitrogen) and Alexa Fluor 594‐conjugated anti‐mouse IgG (1:100 Invitrogen) secondary antibodies were added to the sections to visualize the staining. DAPI (1:200 Invitrogen) was used for nuclear staining. Immunofluorescence images were taken with a Zeiss LSM 880 on an Airyscan confocal microscope (Carl Zeiss, Göttingen, Germany).

The osteogenic ability of each group was shown by observing the formation rate of new mineralized bone. Ten days before sacrifice, mice were given an intraperitoneal injection of Calcein (30 mg/kg Sigma‐Aldrich, St. Louis, MO, USA), and 3 days before sacrifice, they received a dose of Alizarin red (30 mg/kg Sigma‐Aldrich) to form dual fluorescent labels. Sample preparation followed a previously described method [59]. Briefly, femurs were collected, fixed, and subsequently immersed in 5% aqueous potassium hydroxide (KOH) for 96 h. After being embedded in paraffin, each mineralized femur was cut into 5‐µm slices with a microtome and observed with an APX100 microscope (Olympus, Waltham, MA, USA). ImageJ (Version 1.53a, National Institutes of Health, Bethesda, MD, USA) software was used to evaluate the bone formation rate of each bone surface (BFR/BS). Five samples in each group were tested.

### 4.6 Enzyme‐Linked Immunosorbent Assay (ELISA)

Blood was collected from Sham and OVX mice, and the serum was separated. Serum RANKL and OPG were measured using RANKL ELISA assay kits (Abcam) and OPG ELISA assay kits (R&D Systems, MN, USA), respectively, according to each manufacturer's protocols.

### 4.7 Flow Cytometry

We first extracted femoral BMCs without red blood cells and then incubated the resulting single‐cell suspension with 1:200 anti‐CD16/32 (FcγRIII/II Invitrogen) for 15 min to block the nonspecific binding of Fc receptors. Antibody mixtures, CD11b (Invitrogen), HSD17B7 (ProteinTech Group), and ERα (R&D Systems, Minneapolis, MN, USA) typically diluted at 1:100, were added and incubated in the dark at 4°C for 30 min. Subsequently, appropriate secondary antibodies were added and incubated at room temperature for 30 min. Data were acquired with a FACS Aria III (BD, Franklin Lakes, NJ, USA). The analysis was performed using FlowJo software (Version 10.0.7, Tree Star, San Carlos, CA).

### 4.8 Cell Culture, Transient Transfection, and Promoter Luciferase Assay

Human embryonic kidney 293T (HEK293T) cells were obtained from the American Type Culture Collection (Manassas, VA, USA) and cultured in Dulbecco's modified Eagle medium (DMEM). To examine the relationships of HSD17B7, HEK293T cells were transfected with 1 µg of plasmid DNA containing Flag, MYC‐HSD17B7 (#RC209534, OriGene Technologies, Rockville, MD, USA), sh‐HSD17B7 (sc‐88433‐SH, Santa Cruz), or HA‐ERα (Applied Biological Materials Inc., Richmond, Canada) using Lipofectamine 3000 (Invitrogen, Carlsbad, CA, USA). To perform luciferase reporter assay, 1 µg each of FasL and ERE promoter luciferase (Promega, Madison, WI, USA) were used. Briefly, HEK293T cells were transfected with plasmids encoding the Flag, MYC‐HSD17B7, shHSD17B7, ERE‐luc, FasL‐luc, or Renilla luciferase reporter (pRL‐TK‐luc). After 24 more h, the cells were harvested in a reporter lysis buffer. Luciferase activity was determined in whole cell lysates using a luciferase assay kit (Promega, Madison, WI, USA). For electroporation, CD11b^+^ cKO BMCs were used following 2 days of M‐CSF‐induced differentiation. Briefly, 2 × 10^6^ CD11b^+^ cKO BMCs were resuspended in 100 µL of Nucleofector Solution (Mirus Bio, MIR 50117, Madison, WI, USA) and mixed with 2 µg of ERα plasmid. Electroporation was performed using the Amaxa Nucleofector II device (Lonza, Cologne, Germany) with program Y‐001. Cells were immediately transferred into prewarmed medium and cultured for 24 h before subsequent analyses.

### 4.9 Subcellular Fractionation, Coimmunoprecipitation, and Western Blotting

We extracted proteins from tissues or cells using a protein extraction kit (#78510 or 78505, Thermo Fisher Scientific, Waltham, MA, USA) according to the manufacturer's protocol. Nuclear, membrane, and cytoplasmic extracts were isolated using a subcellular protein fractionation kit (#78840, Thermo Fisher Scientific). Homogenates (20 µg for Western blotting) were separated by SDS‐PAGE and transferred to nitrocellulose membranes. For coimmunoprecipitation, 400 µg of homogenates were immunoprecipitated with the indicated antibody at 4°C overnight. The immunocomplexes were pulled down using protein A/G agarose beads (#20421 Thermo Fisher Scientific) and separated by Western blotting. After blocking the samples with 5% skim milk, they were incubated overnight at 4°C with primary antibodies. They were then incubated with HRP‐conjugated secondary antibodies for 1 h at room temperature. The membranes were then visualized using an enhanced chemiluminescence detection kit (Millipore, Billerica, USA). Immunoreactive bands were shown with a LAS‐4000 imager (GE Healthcare Life Science, Pittsburgh, PA, USA). The primary antibodies used for Western blotting were HSD17B7, RANKL (1:1000 Santa Cruz Biotechnology), ERα (R&D Systems), HA, MYC, PLD1, phosphorylated PLD1, mTOR, phosphorylated mTOR, S6K, phosphorylated S6K, S6, phosphorylated S6, ubiquitin (1:1000 Cell Signaling Technology, Danvers, MA, USA), Col1a1, OPN, OPG, total OxPhos, NFATC1 (1:2000 Abcam), and GAPDH (1:2000 Cell Signaling Technology).

### 4.10 Molecular Docking Analysis

We obtained the protein structures of ERα and HSD17B7 from the UniProt database and then processed them using PyMOL (Version 2.1, Schrödinger, Inc., New York, NY, USA) and uploaded them to the HDOCK SERVER website (http://hdock.phys.hust.edu.cn/) for protein–protein docking. We selected the combination with the highest docking score and confidence score for simulating the docking site and used PyMOL for the visual analysis.

For docking with RAL, we obtained the SDF format file of its main active ingredient from the PubChem database. We then collected the protein structures of ERα and HSD17B7 from the PDB database. We used PyMOL to optimize the targets by removing water molecules and small molecule ligands and then used AutoDock Tools (National Institutes of Health, Bethesda, MD, USA) to add hydrogen atoms and charge treatments, saving the files in pdbqt format. We then performed molecular docking using PyRx (version 0.9.7) software's internal vina, calculated the binding energy, and output the result files. We used PyMOL for result visualization.

### 4.11 Osteogenic Function Analysis

As previously described, primary osteoblasts were extracted from mouse femurs and cultured in osteogenic induction medium (MUXMX‐90021 Cyagen) for 7 or 14 days [60]. The cells were fixed with 4% paraformaldehyde for 10 min. For alkaline phosphatase (ALP) staining, the 7‐day cells were treated with a BCIP/NBT kit (K4151 ApexBio, Houston, USA). The stained images were captured with a digital camera and quantified by measuring the OD value at 405 nm with an ALP assay kit (ab83369 Abcam). For Alizarin red S (ARS) staining, cells cultured for 14 days were treated with 40 mM ARS (Sigma‐Aldrich), pH 4.0, and the staining results were photographed. Subsequently, 10% chlorinated pivaloyl chloride (Sigma‐Aldrich) was added, and quantification was performed by measuring the OD value at 560 nm.

### 4.12 Mutagenesis of HSD17B7

Based on the docking prediction results for HSD17B7 and ERα, corresponding deletion mutants from the HSD17B7‐MYC plasmid were generated (Gene Synthesis, Seoul, Korea). Specifically, HSD17B7^d5–45^, HSD17B7^d119–172^, HSD17B7^d192–200^, and HSD17B7^d274–302^ mutants were generated by deleting the corresponding sequences starting from the N‐terminal sequence of HSD17B7.

### 4.13 Real‐Time PCR With Reverse‐Transcription Analysis

Total RNA was extracted using TRIzol reagent (Invitrogen, Life Technologies, Carlsbad, CA, USA) according to the manufacturer's instructions. The extracted total RNA sample (1 µg) was reverse transcribed into cDNA using a first‐strand cDNA synthesis kit (Applied Biosystems, Foster City, CA, USA). Quantitative PCR was performed in 384‐well plates using the ABI Prism 7900HT Sequence Detection System (Applied Biosystems, Foster City, CA, USA). RNA from each sample was analyzed individually. The primer sequences used for PCR are listed in Table S2.

### 4.14 Measurement of the Oxygen Consumption Rate

OCR was measured using an XF24 extracellular analyzer (Agilent Technologies, Santa Clara, CA, USA). CD11b^+^ BMCs (2 × 10^5^ cells per well) treated with M‐CSF and RANKL for 3 days were seeded in 24‐well plates. The following day, the medium was replaced with assay medium containing 10 mM glucose, 1 mM pyruvate, and 2 mM glutamine. Cells were cultured for 1 h in a CO‐free incubator at 37°C and then sequentially exposed to oligomycin (1 µM), FCCP (1 µM), and rotenone (0.5 µM), included in the Seahorse XF Cell Mito Stress Kit (Agilent Technologies). Data were processed using Wave software (Agilent Technologies).

### 4.15 TUNEL Assay and Assessment of Intracellular Reactive Oxygen Species

Following the manufacturer's instructions, a DeadEnd colorimetric TUNEL system (G7360, Promega Madison, WI, USA) was used to assess apoptosis in CD11b^+^ osteoclasts. The nuclei were stained with hematoxylin. To measure intracellular ROS levels, CD11b^+^ osteoclasts were incubated with 10 mM H2DCFDA (D399, Life Technologies) for 30 min. The nuclei were stained with Hoechst (HY‐15559, MedChemExpress, Monmouth Junction, NJ, USA). Observations and five to six microscopic images were obtained using an APX100 microscope. ImageJ (Version 1.53a) software was then used to evaluate the ratio of positive cells to total cells in each field of view.

### 4.16 Evaluation of Mitochondrial Membrane Potential

CD11b^+^ osteoclasts were incubated with JC‐10 (786‐1549 G‐Biosciences, St. Louis, USA) dissolved in DMEM for 30 min. We used the APX100 microscope to observe JC‐10 polymers (Ex/Em = 540/590 nm) and JC‐10 monomers (Ex/Em = 490/525 nm). ImageJ (Version 1.53a) software was used to evaluate the ratio of JC‐10 polymer cells to JC‐10 monomers cells in each field of view.

### 4.17 Transmission Electron Microscopy and Image Analysis

To compare the mitochondrial morphology of WT mice and HSD17B7^Oc^ KO mice, CD11b^+^ BMCs treated with M‐CSF and RANKL for 3 days were collected and observed using transmission electron microscopy (TEM). As previously described [61], the samples were fixed, embedded, sectioned, and then observed and imaged using a Hitachi Bio‐TEM (H‐7650, Hitachi, Tokyo, Japan). Based on previous research [62], ImageJ (Version 1.53a) and DigitalMicrograph software (Version 3.6, Gatan Microscopy Suite, USA) were used to calculate the number of mitochondria.

### 4.18 RNA Sequencing Data Analysis

Mouse femur BMCs from control mice (*n* = 4) and cKO mice (*n* = 4) were extracted and differentiated into preosteoclasts (1 × 10^6^ cells) for RNA sequencing. Total RNA was isolated from WT and cKO preosteoclasts using an RNeasy mini kit (QIAGEN Valencia, CA, USA) with DNase treatment to remove genomic DNA contamination. Strand‐specific RNA libraries were prepared using a KAPA mRNA HyperPrep kit (KAPA Biosystems, Foster City, CA, USA) according to the manufacturer's protocol. The prepared libraries were sequenced on an Illumina HiSeq 4000 instrument (Illumina, San Diego, CA, USA) to generate paired‐end reads. Raw sequencing data were processed using standard quality control procedures to remove adapters and low‐quality bases. The processed data were aligned to the mouse reference genome using StringTie, which allowed for the estimation of gene abundance from read counts. Differential gene expression analysis was performed using the DESeq2 package within the R statistical environment (version 4.3.2). Genes with an adjusted *p*‐value < 0.05 were considered significantly differentially expressed. Gene enrichment analysis, functional annotation analysis, and pathway analysis for the list of significant genes were performed using the g: Profiler pathway (https://biit.cs.ut.ee/gprofiler/).

### 4.19 Statistical Analysis

Data are presented as the mean ± standard error of the mean (SEM). Statistical comparisons were performed using one‐way analysis of variance (ANOVA) followed by the Tukey post hoc test. The significance of differences between groups was determined using Student's independent *t*‐test. Linear regression analysis was performed using GraphPad Prism version 9.00 (San Diego, CA, USA). A *p*‐value < 0.05 was considered statistically significant.

## Author Contributions

Junyue Zhang, Yiping Song, Jeong‐Hyun Koo, and Si Chen performed experiments and analyzed the data. Young Jae Moon designed experiment. Sun‐Jung Yoon provided the clinical samples. Junyue Zhang and Young Jae Moon wrote the manuscript. Kyu Yun Jang and Jung Ryul Kim supervised and conceived the project. All the authors critically revised and approved the manuscript.

## Funding

This work was supported by a grant from the National Research Foundation (NRF‐2022R1C1C1006721, 2022R1A2C2005734, RS‐2023‐00236157), a grant from the Korea Health Technology R&D Project through the Korea Health Industry Development Institute (KHIDI), funded by the Ministry of Health & Welfare (HR22C1832, RS‐2024‐00335828), by Fund of Biomedical Research Institute, Jeonbuk National University Hospital.

## Ethics Statement

The animal study protocol was approved by the Institutional Animal Care and Use Committee of Jeonbuk National University Hospital (Permit No: JBUH‐IACUC‐2020‐20). The human study was performed with the approval of the institutional review board at Jeonbuk National University Hospital (IRB No: JBUH 2023‐12‐023) and conformed to the Declaration of Helsinki.

## Conflicts of Interest

The authors declare no conflicts of interest.

## Supporting information




**TABLE S1** Primers used for genotyping mice.
**TABLE S2** The primer sequences used in the PCR.
**FIGURE S1** FACS analysis of HSD17B7 and ERα in CD11b+ bone marrow cells from Sham/OVX and young/old mice.
**FIGURE S2** HEK293T cells were transfected with empty vector or shRNA for HSD17B7. Twenty‐four hours after transfection, the cells were treated with 20 µg/mL cycloheximide (CHX) or 20 µM MG132 for 0.5–2 h. The cell lysates were blotted with GAPDH antibody, and the relative protein levels of ERα were compared (*n* = 3).
**FIGURE S3** After comparing the amino acid sequences of HSD17B7 across seven known species, glutamate(E) 121 was highly conserved. A docking module analysis of human ERα and HSD17B7 revealed potential docking sites.
**FIGURE S4** After transfection in HEK293T cells with ERα, HSD17B7, or shHSD17B7, an immunoprecipitation assay using ERα antibody to determine the physical interaction between CHIP or USP7 and ERα was performed.
**FIGURE S5** Lacking HSD17B7 does not affect osteoclastogenic cytokines, osteoblast function, and cortical bone phenotype. (A) Six weeks after surgery, uterus weight was determined (*n* = 3–4). (B, C) Primary bone marrow stromal cells (BMSCs) were extracted from femurs and cultured in osteogenic induction medium (MUXMX‐90021 Cyagen) for 4 days. The expression and secretion of RANKL and OPG were confirmed using Western blot analysis of cell lysates (B) and an ELISA of supernatants (C). (D) Primary osteogenic differentiated BMSCs conditioned media (CM) from WT and HSD17B7^+/−^ and RANKL were used to treat WT CD11b^+^ cells to induce osteoclastogenesis, and TRAP staining was performed. Representative TRAP staining after osteoclastogenesis induction. Scale bars = 100 µm. TRAP‐positive multinucleate cells with three or more nuclei were counted as osteoclasts and were scored per field (*n* = 5). (E) qPCR for osteoblast marker genes and osteoclastogenic cytokines in primary osteoblasts cells (*n* = 3–4). (F, G) Representative micro‐CT reconstruction of cortical bone and micro‐CT analysis. Ct.Ar, cortical area; Ct.Th, cortical thickness; Ec.Pm, endosteal perimeter; pMOI, polar moment of inertia; Ps.Pm, periosteal perimeter; TMD, tissue mineral density. *p < 0.05 versus WT or WT Sham.
**FIGURE S6** Generation of monocyte‐specific HSD17B7 knockout mice and RANKL‐activated signaling pathway changes. (A) Mating schematic diagram for HSD17B7 knockout (cKO) mice. PCR was used to identify the genotypes of the *Hsd17b7^fl/fl^* and *Lyz2‐Cre* mice. (B) Western blots of multiorgan protein expression from WT and cKO mice. (C) HSD17B7 has no effect on the regulation of RANKL‐activated signaling pathways. CD11b^+^ WT/cKO BMCs were treated with M‐CSF (10 ng/mL) and sRANKL (30 ng/mL) for the indicated periods. Cell lysates were prepared. Changes in MAPKs, Akt, and NF‐κB were evaluated by Western blotting, and the results were quantified (*n* = 3). Values presented are the mean ± SEM. **p < 0.01 versus WT.
**FIGURE S7** CD11b^+^ Control/cKO BMCs were treated with VU0359595 (1 µM) for 24 h. The expression patterns of the PLD1–mTOR signaling pathway and OxPhos complex were determined via Western blotting, and quantization results of Figure 6H (*n* = 3).
**FIGURE S8** The docking module of HSD17B7 and raloxifene was predicted by AutoDock and exhibited with PyMol. (We suspected that HSD17B7 could also combine with raloxifene. To explore that hypothesis, we used AutoDock Vina to simulate molecular docking between HSD17B7 and raloxifene, and the binding energy was ‐8.5 kcal/mol, indicating a strong potential for real binding.)

## Data Availability

All data supporting the findings of this study are available within the article and its supporting information files. RNA‐seq data will be deposited in NCBI Gene Expression Omnibus database (GSE274826) and will be publicly available from the date of publication.
