# Supplementary material for: HSD17B7 Counters Bone Loss in Estrogen Deficiency via Estrogen Receptor Stabilization and Mediates the Effect of Raloxifene
Source: MedComm (2020). 2026 Jan 31;7(2):e70623. doi: 10.1002/mco2.70623 (PMC12860897; doi:10.1002/mco2.70623)
Supplement: Supplementary file 1 — TABLE S1 Primers used for genotyping mice. TABLE S2 The primer sequences used in the PCR. FIGURE S1 FACS analysis of HSD17B7 and ERα in CD11b+ bone marrow cells from Sham/OVX and young/old mice. FIGURE S2 HEK293T cells were transfected with empty vector or shRNA for HSD17B7. Twenty‐four hours after transfection, the cells were treated with 20 µg/mL cycloheximide (CHX) or 20 µM MG132 for 0.5–2 h. The cell lysates were blotted with GAPDH antibody, and the relative protein levels of ERα were compared (n = 3). FIGURE S3 After comparing the amino acid sequences of HSD17B7 across seven known species, glutamate(E) 121 was highly conserved. A docking module analysis of human ERα and HSD17B7 revealed potential docking sites. FIGURE S4 After transfection in HEK293T cells with ERα, HSD17B7, or shHSD17B7, an immunoprecipitation assay using ERα antibody to determine the physical interaction between CHIP or USP7 and ERα was performed. FIGURE S5 Lacking HSD17B7 does not affect osteoclastogenic cytokines, osteoblast function, and cortical bone phenotype. (A) Six weeks after surgery, uterus weight was determined (n = 3–4). (B, C) Primary bone marrow stromal cells (BMSCs) were extracted from femurs and cultured in osteogenic induction medium (MUXMX‐90021 Cyagen) for 4 days. The expression and secretion of RANKL and OPG were confirmed using Western blot analysis of cell lysates (B) and an ELISA of supernatants (C). (D) Primary osteogenic differentiated BMSCs conditioned media (CM) from WT and HSD17B7+/− and RANKL were used to treat WT CD11b+ cells to induce osteoclastogenesis, and TRAP staining was performed. Representative TRAP staining after osteoclastogenesis induction. Scale bars = 100 µm. TRAP‐positive multinucleate cells with three or more nuclei were counted as osteoclasts and were scored per field (n = 5). (E) qPCR for osteoblast marker genes and osteoclastogenic cytokines in primary osteoblasts cells (n = 3–4). (F, G) Representative micro‐CT reconstruction of cortical bone an [file MCO2-7-e70623-s001.docx]

**HSD17B7 counters bone loss in estrogen deficiency via estrogen receptor stabilization and mediates the effect of raloxifene**

**Supplemental information**

**1. Supplemental table**

**2. Supplemental figures**

**1. Supplemental table**

**Table S1.** Primers used for genotyping mice

| Name | Primer Sequence | |
| --- | --- | --- |
| Hsd17b7^fl/fl^ | forward | 5’-TGACACATATTCTGAAGCAAGCCT-3’ |
|  | reverse | 5’-ATCTACCAACACAGAAGACCCAAG-3’ |
| Hsd17b7^+/-^ | forward | 5′-CACATAGTAGCTAAAGCAGCAACA-3′ |
|  | reverse | 5′-CAAATACAGGTTCTACGCAAGCAA-3′ |
| LysM-Cre | Mutant Reverse | 5′-CCCAGAAATGCCAGATTACG-3′ |
|  | Common | 5′-CTTGGGCTGCCAGAATTTCTC-3′ |
|  | Wild type Reverse | 5′-TTACAGTCGGCCAGGCTGAC-3′ |

**Table S2.** The primer sequences used in the PCR

| **qRT-PCR** | | |
| --- | --- | --- |
| Genes | Forward (5′ to 3′) | Reverse (3′ to 5′) |
| *Hsd17b7* | CTGTGACACCGTACAACGGA | GCTCGGGTGATCCGATTTCT |
| *Nrf1* | AGCACGGAGTGACCCAAAC | TGTACGTGGCTACATGGACCT |
| *Sdhb* | AATTTGCCATTTACCGATGGGA | AGCATCCAACACCATAGGTCC |
| *Tfam* | GGAATGTGGAGCGTGCTAAAA | ACAAGACTGATAGACGAGGGG |
| *Mcad* | AGGGTTTAGTTTTGAGTTGACGG | CCCCGCTTTTGTCATATTCCG |
| *Cycs* | CCAAATCTCCACGGTCTGTTC | ATCAGGGTATCCTCTCCCCAG |
| *Ndufs1* | TGCAAATCCCTCGATTCTGTTAC | GCTTTCTCAATCTCTACCAGGC |
| *Ndufv2* | GCAAGGAATTTGCATAAGACAGC | TAGCCATCCATTCTGCCTTTG |
| *Trap* | CAGCAGCTCCCTAGAAGATGG | CTGGAACCTCTTGTCGCTGG |
| *CathpsinK* | GAAGAAGACTCACCAGAAGCAG | TCCAGGTTATGGGCAGAGATT |
| *Nfatc1* | GACCCGGAGTTCGACTTCG | TGACACTAGGGGACACATAACTG |
| *Col1a* | GTGCTAAAGGTGCCAATGGT | ACCAGGTTCACCGCTGTTAC |
| *Spp1* | ATCTCACCATTCGGATGAGTCT | TGTAGGGACGATTGGAGTGAAA |
| *Osx* | TTACAAGCACTAATGGGCTCCT | GTAGACACTGGGCAGACAGTCA |
| *Bglap* | CTGACCTCACAGATCCCAAGC | TGGTCTGATAGCTCGTCACAAG |
| *Runx2* | TTACTTACACCCCGCCAGTC | CACTCTGGCTTTGGGAAGAG |
| *Tnfsf11* | CAGCATCGCTCTGTTCCTGTA | CTGCGTTTTCATGGAGTCTCA |
| *Tnfrsf11b* | ACCCAGAAACTGGTCATCAGC | CTGCAATACACACACTCATCACT |
| *Mtco1* | CTACTATTCGGAGCCTGAGC | GCATGGGCAGTTACGATAAC |
| *Mtco2* | AACCATAGGGCACCAATGATAC | GGATGGCATCAGTTTTAAGTCC |
| *Cox5b* | TTCAAGGTTACTTCGCGGAGT | CGGGACTAGATTAGGGTCTTCC |
| *Ppia* | GAGCTGTTTGCAGACAAAGTTC | CCCTGGCACATGAATCCTGG |
| *Rps3* | CAGGCAGAGTCTCTACGCTACA | TCTCCATAATGAACCGAAGCA |
| *Gapdh* | CAACTCCCTCAAGATTGTCAGCAA | GGCATGGACTGTGGTCATGA |

**2. Supplemental figures**

**
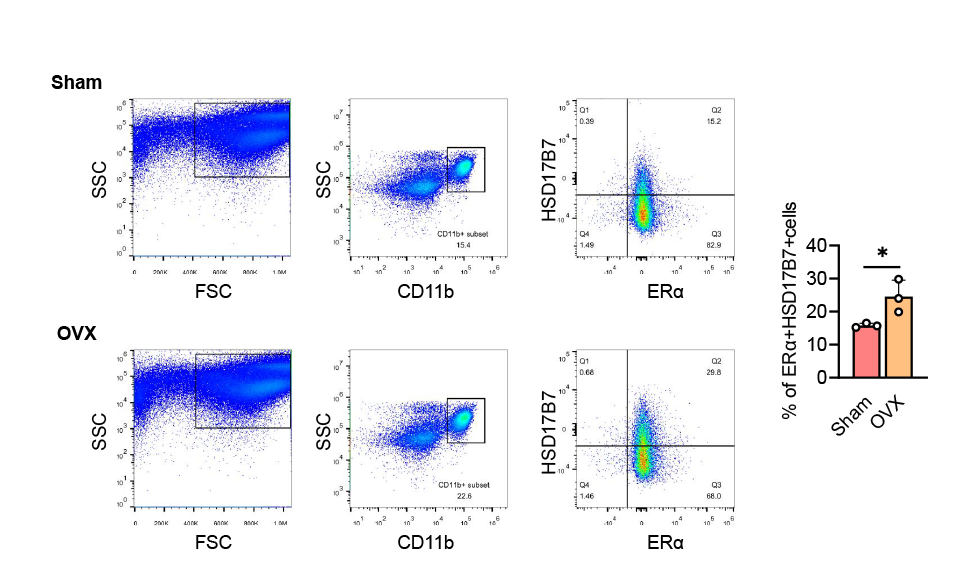
**

**Figure S1** FACS analysis of HSD17B7 and ERα in CD11b+ bone marrow cells from Sham/OVX and Young/Old mice.


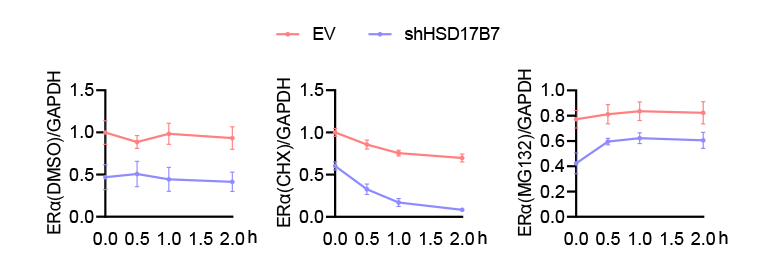


**Figure S2** HEK293T cells were transfected with empty vector or shRNA for HSD17B7. 24 h after transfection, the cells were treated with 20 μg/ml cycloheximide (CHX) or 20 μM MG132 for 0.5 to 2 h. The cell lysates were blotted with GAPDH antibody, and the relative protein levels of ERα were compared (n = 3).

**
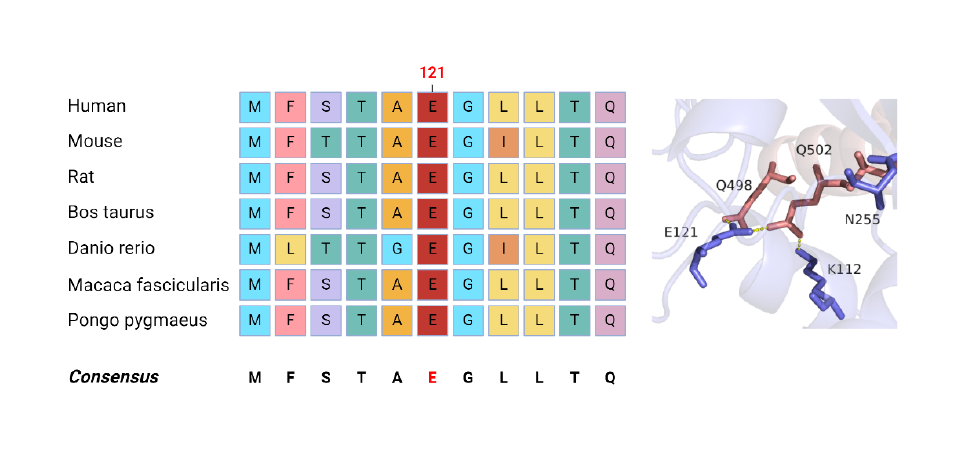
**

**Figure S3** After comparing the amino acid sequences of HSD17B7 across seven known species, glutamate(E) 121 was highly conserved. A docking module analysis of human ERα and HSD17B7 revealed potential docking sites.


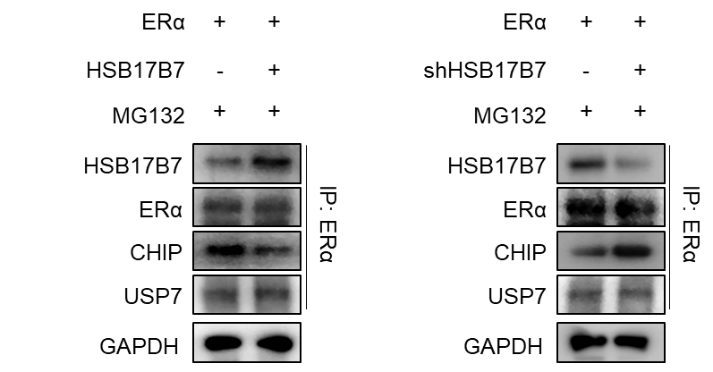


**Figure S4** After transfection in HEK293T cells with ERα, HSD17B7 or shHSD17B7, an immunoprecipitation assay using ERα antibody to determine the physical interaction between CHIP or USP7 and ERα was performed.


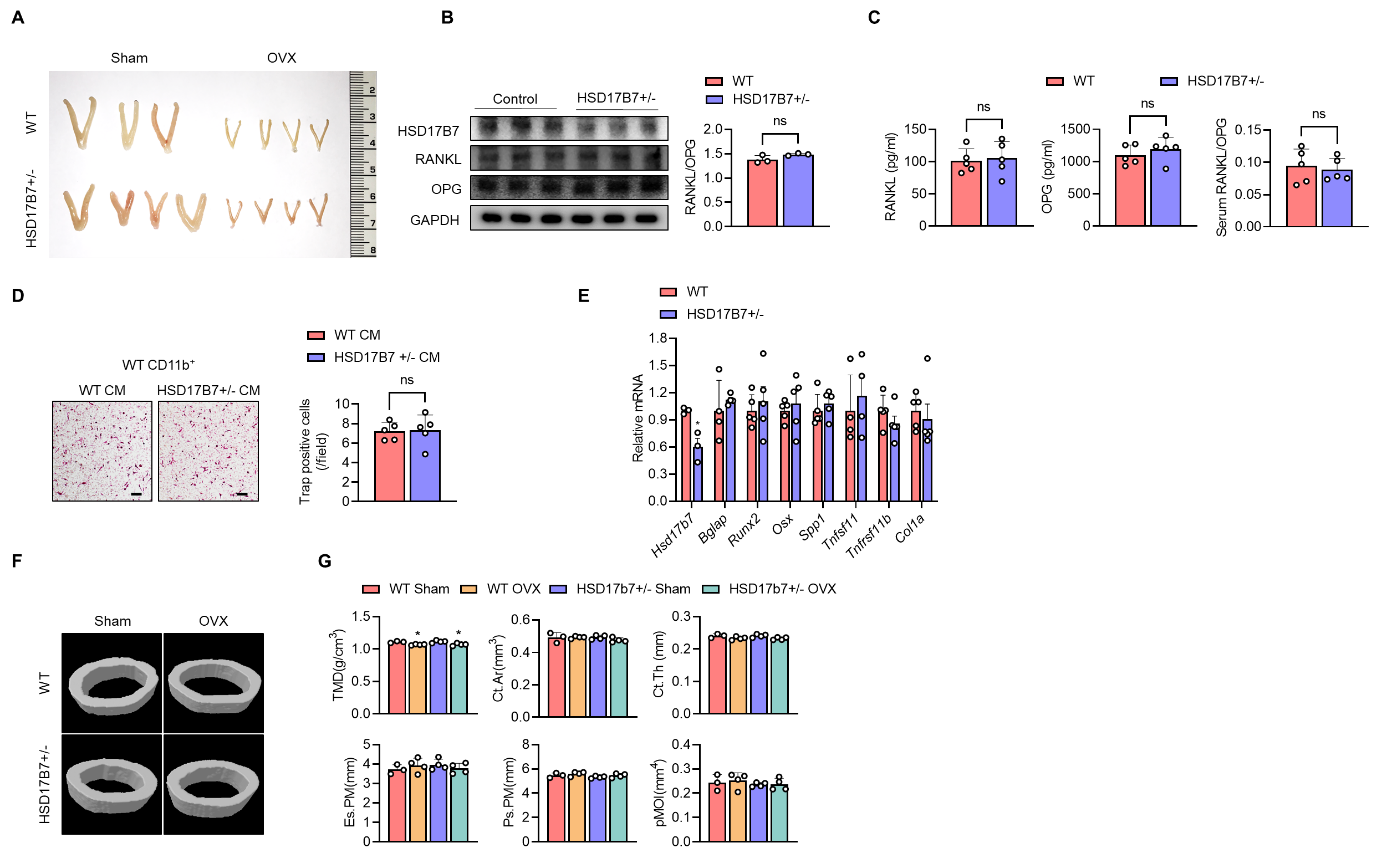


**Figure S5 Lacking HSD17B7 does not affect osteoclastogenic cytokines,** **osteoblast function, and cortical bone phenotype.** (A) Six weeks after surgery, uterus weight was determined (n = 3–4). (B-C) Primary bone marrow stromal cells (BMSCs) were extracted from femurs and cultured in osteogenic induction medium (MUXMX-90021 Cyagen) for 4 days. The expression and secretion of RANKL and OPG were confirmed using Western blot analysis of cell lysates (B) and an ELISA of supernatants (C). (D) Primary osteogenic differentiated BMSCs conditioned media (CM) from WT and HSD17B7^+/-^ and RANKL were used to treat WT CD11b^+^ cells to induce osteoclastogenesis, and TRAP staining was performed. Representative TRAP staining after osteoclastogenesis induction. Scale bars = 100 µm. TRAP-positive multinucleate cells with three or more nuclei were counted as osteoclasts and were scored per ﬁeld (n = 5). (E) qPCR for osteoblast marker genes and osteoclastogenic cytokines in primary osteoblasts cells (n = 3–4). (F, G) Representative micro-CT reconstruction of cortical bone and micro-CT analysis. TMD: tissue mineral density; Ct.Ar: cortical area; Ct.Th: cortical thickness; Ec.Pm: endosteal perimeter; Ps.Pm: periosteal perimeter; pMOI: Polar moment of inertia.*p < 0.05 versus WT or WT Sham.


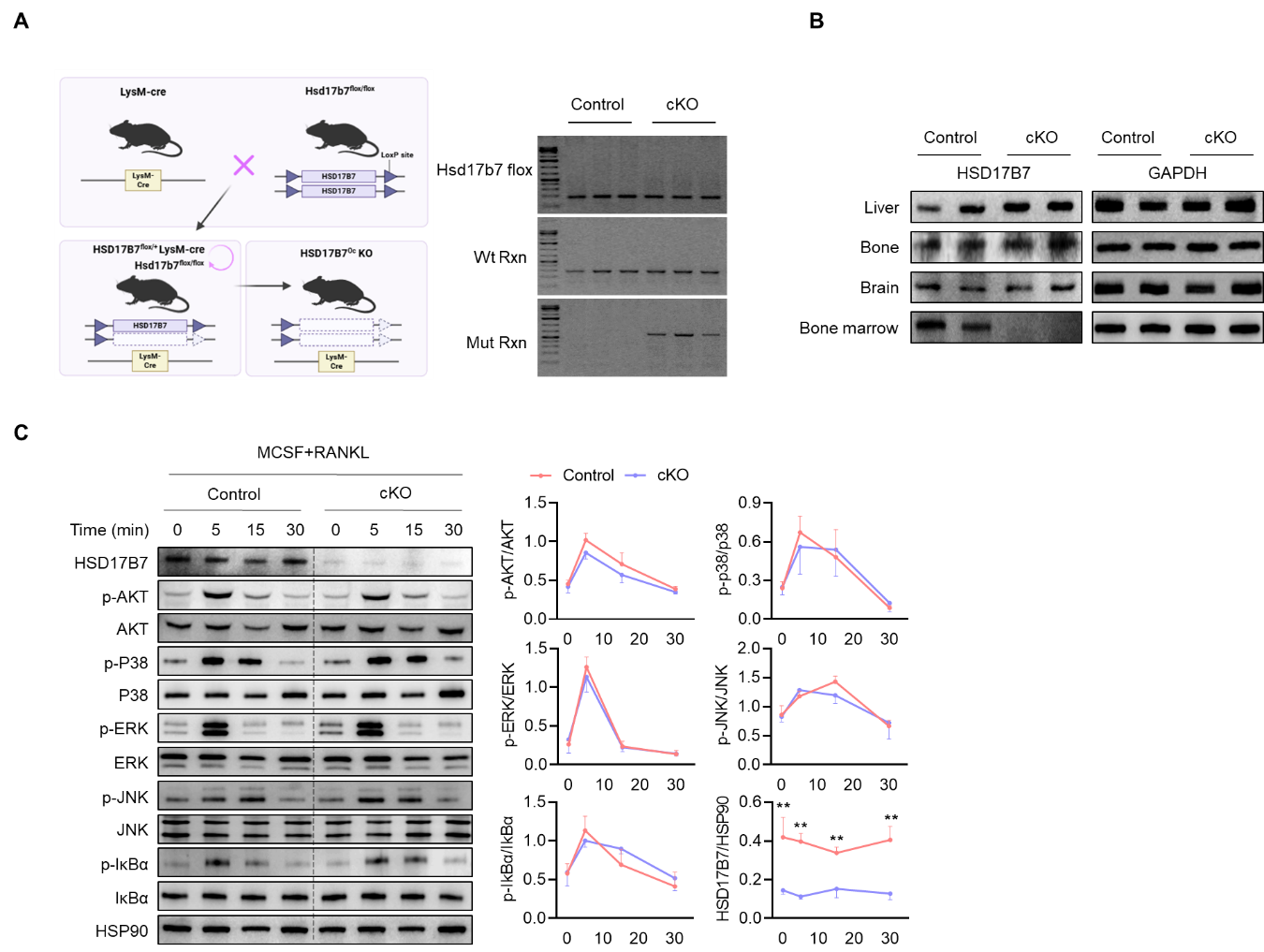


**Figure S6 Generation of monocyte-specific HSD17B7 knockout mice and RANKL-activated signaling pathway changes.** (A) Mating schematic diagram for HSD17B7 knockout (cKO) mice. PCR was used to identify the genotypes of the *Hsd17b7^fl/fl^* and *Lyz2-Cre* mice. (B) Western blots of multi-organ protein expression from WT and cKO mice. (C) HSD17B7 has no effect on the regulation of RANKL-activated signaling pathways. CD11b^+^ WT/cKO BMCs were treated with M-CSF (10 ng/ml) and sRANKL (30 ng/ml) for the indicated periods. Cell lysates were prepared. Changes in MAPKs, Akt, and NF-κB were evaluated by Western blotting, and the results were quantified (n = 3). Values presented are the mean ± SEM. **p < 0.01 versus WT.


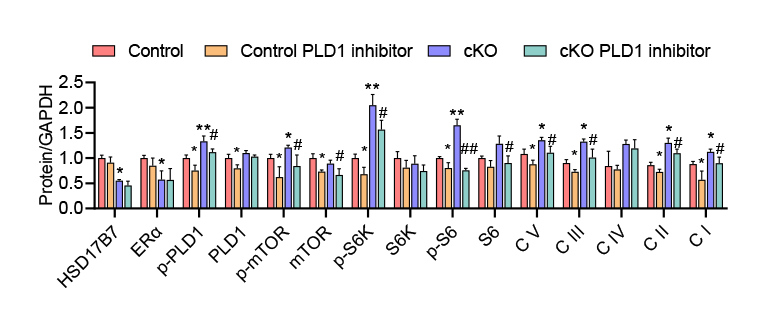


**Figure S7** CD11b^+^ Control/cKO BMCs were treated with VU0359595 (1 μM) for 24 h. The expression patterns of the PLD1-mTOR signaling pathway and OxPhos complex were determined via Western blotting, and quantization results of Fig 6h (n = 3).


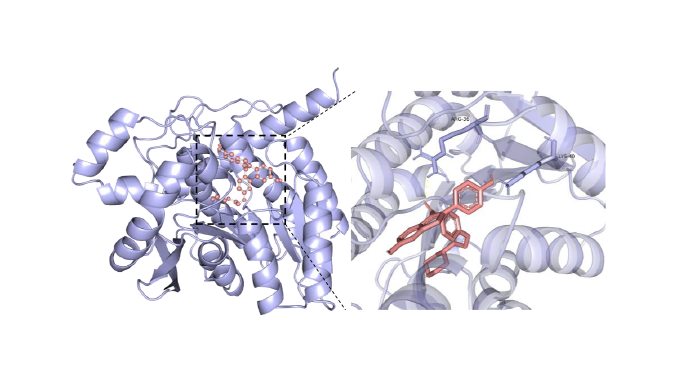


**Figure S8** The docking module of HSD17B7 and raloxifene was predicted by AutoDock and exhibited with PyMol. (We suspected that HSD17B7 could also combine with raloxifene. To explore that hypothesis, we used AutoDock Vina to simulate molecular docking between HSD17B7 and raloxifene, and the binding energy was -8.5 kcal/mol, indicating a strong potential for real binding.)
